# Development of tools for the genetic manipulation of *Campylobacter* and their application to the *N-*glycosylation system of *Campylobacter hepaticus,* an emerging pathogen of poultry

**DOI:** 10.1128/mbio.01101-24

**Published:** 2024-07-29

**Authors:** Jamieson B. McDonald, Ben Wade, Daniel M. Andrews, Thi Thu Hao Van, Robert J. Moore

**Affiliations:** 1 School of Science, RMIT University, Bundoora West Campus, Bundoora, Victoria, Australia; 2 Bioproperties Pty Ltd, RMIT University, Bundoora West Campus, Bundoora, Victoria, Australia; LMU Munich, Munich, Germany

**Keywords:** *Campylobacter hepaticus*, spotty liver disease, mutagenesis, plasmid, shuttle vectors, *N-*glycosylation

## Abstract

Various species of campylobacters cause significant disease problems in both humans and animals. The continuing development of tools and methods for genetic and molecular manipulation of campylobacters enables the detailed study of bacterial virulence and disease pathogenesis. *Campylobacter hepaticus* is an emerging pathogen that causes spotty liver disease (SLD) in poultry. SLD has a significant economic and animal welfare impact as the disease results in elevated mortalities and significant decreases in egg production. Although potential virulence genes of *C. hepaticus* have been identified, they have not been further studied and characterized, as appropriate genetic tools and methods to transform and perform mutagenesis studies in *C. hepaticus* have not been available. In this study, the genetic manipulation of *C. hepaticus* is reported, with the development of novel plasmid vectors, methods for transformation, site-specific mutagenesis, and mutant complementation. These tools were used to delete the *pglB* gene*,* an oligosaccharyltransferase, a central enzyme of the *N-*glycosylation pathway, by allelic exchange. In the mutant strain, *N*-glycosylation was completely abolished. The tools and methods developed in this study represent innovative approaches that can be applied to further explore important virulence factors of *C. hepaticus* and other closely related *Campylobacter* species.

**IMPORTANCE:**

Spotty liver disease (SLD) of layer chickens, caused by infection with *Campylobacter hepaticus*, is a significant economic and animal welfare burden on an important food production industry. Currently, SLD is controlled using antibiotics; however, alternative intervention methods are needed due to increased concerns associated with environmental contamination with antibiotics, and the development of antimicrobial resistance in many bacterial pathogens of humans and animals. This study has developed methods that have enabled the genetic manipulation of *C. hepaticus*. To validate the methods, the *pglB* gene was inactivated by allelic exchange to produce a *C. hepaticus* strain that could no longer *N*-glycosylate proteins. Subsequently, the mutation was complemented by reintroduction of the gene in *trans*, on a plasmid vector, to demonstrate that the phenotypic changes noted were caused by the mutation of the targeted gene. The tools developed enable ongoing studies to understand other virulence mechanisms of this important emerging pathogen.

## INTRODUCTION

*Campylobacter hepaticus* is an emerging bacterial pathogen that causes spotty liver disease (SLD) in layer hens. It has been reported in many regions of the world and represents a significant economic and animal welfare threat to the global poultry industry (1–6). Layer birds infected with *C. hepaticus*, especially free-range hens, can develop numerous hepatic lesions during the peak time of lay (6). SLD can increase flock mortality rates by up to 15% (7–9) and decrease egg production by up to 35% (10). More recently, a second novel *Campylobacter* species, *Campylobacter bilis,* was isolated from the bile of SLD-positive birds (11) and proven to be an additional cause of SLD (12). The pathogenic mechanisms of *C. hepaticus and C. bilis* remain largely unknown. Therefore, there are currently no effective, targeted interventions against SLD, and prevention and treatment of the disease rely on strict biosecurity measures, which are generally inadequate. The use of antibiotics can be effective (13); however, there are public health concerns associated with antibiotic resistance. In addition, tetracycline-resistant *C. hepaticus* strains have recently been isolated from clinical cases of SLD (13, 14). Therefore, alternative methods to control SLD are needed.

Like the closely related species *Campylobacter jejuni* and *Campylobacter coli, C. hepaticus* colonizes the gastrointestinal tract of chickens (5). However, it is thought that an important mechanism of disease is the ability of *C. hepaticus* to traffic from the gastrointestinal tract to the gall bladder, where it is found in the bile and can induce disease in the liver (13). Transcriptomic analysis of *C. hepaticus* isolated from the bile of SLD positive birds has provided insights into genes, which may be of importance for survival and colonization and hence pathogenesis (14). Several genes predicted to be associated with niche adaptation and virulence were identified, including genes responsible for chemotaxis, motility, lipooligosaccharide synthesis, and metabolism (14). However, phenotypic analysis of these putative virulence mechanisms remains lacking due to inadequate genetic tools to perform mutagenesis studies. Therefore, the precise roles these genes and their products play in the development of SLD have not yet been determined.

Despite reported difficulties in cloning *Campylobacter* DNA and transforming *Campylobacter* (15–17), genetic tools, including shuttle vectors (17–21) and suicide vectors (22–26), have been paramount in understanding the mechanisms of pathogenesis in the closely related species *C. jejuni,* an important human pathogen (27). The successful development of vectors has primarily relied on the utilization of native plasmid DNA elements from *C. jejuni* or closely related species. *C. jejuni* tends to be more efficiently transformed with DNA isolated from the same or closely related species and, in some cases, cannot be transformed with DNA isolated from *Escherichia coli* (17, 19, 28).

Several groups have engineered *E. coli-C. jejuni* shuttle vectors (17–21), usually derivatives of the *E. coli-Campylobacter shuttle* vector, pILL550 (18). This shuttle vector incorporated the *Campylobacter* plasmid replication functions, including an origin of replication and replication proteins, from a high copy number *C. coli* cryptic plasmid. The transformation efficiencies of these vectors vary significantly between *C. jejuni* strains and between different laboratories (29). Thus, groups have characterized cryptic plasmids from *C. jejuni* clinical isolates to develop new expression vectors (29, 30). For example, one study could not successfully transform pILL550 into several strains of *Campylobacter*; hence, they isolated and characterized a *C. jejuni* cryptic plasmid and developed a shuttle vector utilizing the cryptic plasmids replication elements (30). This has proven a reliable means to produce new *E. coli-Campylobacter* shuttle vectors.

**Fig 1:**
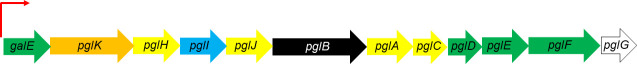
The genetic organization of *C. hepaticus* HV10^T^ protein *N*-glycosylation locus (38). Open reading frames predicted to be involved in sugar biosynthesis are illustrated in green, flippase in orange, glycosyltransferases in yellow, oligosaccharyltransferase in black, and *pglG* in white, as it has no known function in the *C. jejuni N-*glycosylation pathway (39). The red arrow represents the predicted putative promoter of *galE*.

The functionality of these shuttle vectors as tools to genetically complement-inactivated chromosomal genes in *C. jejuni* has played crucial roles in the elucidation of key virulence factors, including the *N-*glycosylation of proteins. The *N-*glycosylation system in *C. jejuni* is encoded by a protein glycosylation (*pgl*) gene locus comprising genes responsible for the biosynthesis and transfer of a conserved heptasaccharide *N-*glycan onto several proteins (31–34). Disruption of the *N-*glycosylation pathway in *C. jejuni* negatively impacts proteome stability and other important enzymatic and cellular pathways leading to significant changes in cell physiology (35, 36). Importantly, this reduces the ability of *C. jejuni* to adhere to and invade human intestinal cells (37), and *C. jejuni* completely lacking *N*-glycoproteins is unable to colonize the gastrointestinal tract of its natural chicken host (35). Recently, *C. hepaticus* was shown to possess an *N-*glycosylation system (Fig. 1), much like *C. jejuni,* which is suspected to play key roles in host colonization, invasion, and pathogenicity (38). However, the further investigation of this system’s function was hampered by the lack of genetic manipulation tools and methods for *C. hepaticus*.

This study aimed to develop genetic tools and methods and reports the genetic manipulation of *C. hepaticus* by the construction of a gene knockout mutant. *E. coli-C. hepaticus* shuttle vectors have been constructed and used to develop a transformation method. These newly developed methods and vectors were used to delete a gene by allelic exchange and complement the mutation. These tools were used to investigate the impacts of *pglB* deletion on the ability of *C. hepaticus* HV10^T^ to *N*-glycosylate proteins.

## RESULTS

### Sequencing of *C. coli* cryptic plasmids pWestmill 11, pCC311, and pCC388

**Fig 2:**
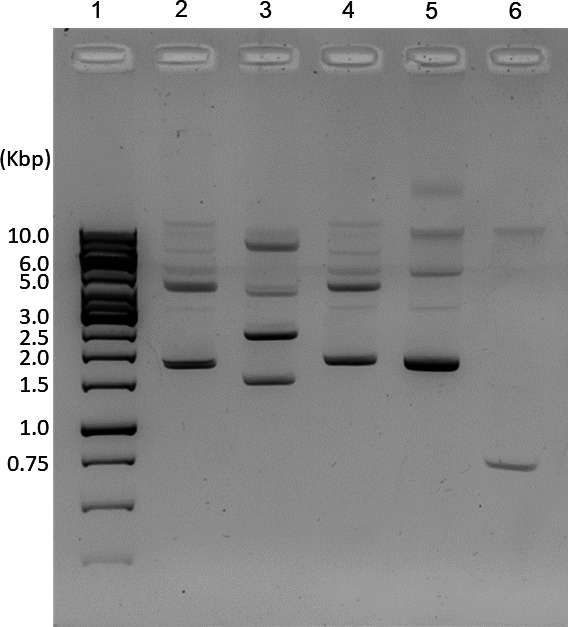
Plasmid DNA isolated from *C. coli* strains. Plasmid extracts were analyzed by electrophoresis on a 1.0% agarose gel. Lanes: 1, GeneRuler 1 kb DNA ladder; 2, *C. coli* 320; 3, *C. coli* 311; 4, *C. coli* 320 #2; 5, *C. coli* 388; 6, *C. coli* Westmill 11.

To obtain replication elements that could be utilized by *C. hepaticus* to functionally maintain and replicate plasmid DNA, multiple *C. hepaticus, C. jejuni,* and *C. coli* strains were screened for low molecular weight plasmids (<10 kb). While no *C. hepaticus* and *C. jejuni* strains contained small plasmids (not shown), several *C. coli* strains contained low molecular weight plasmids (Fig. 2).

Gel extracted plasmid DNAs were sequenced and analyzed for each of these strains. Analysis of DNA sequences revealed that *C. coli* 311, *C. coli* 388, and *C. coli* Westmill 11 all contained at least two plasmids. Blastn analysis of the nucleotide sequence of each plasmid revealed varying levels of sequence similarity to previously identified *C. coli* plasmids (Table 1.)

**TABLE 1:** Sequenced plasmids isolated from different *C. coli* strains

Strain	Plasmid size (bp)	Query cover (%)	Sequence identity (%)	Previously identified *C. coli* plasmid (accession)
311	2,744	75	91.11	X82079
	4,367	100	100	NZ_CP092028
388	26,670	96	98.35	NZ_MH634990
	3,303	100	97.55	DQ518171
Westmill 11	27,860	95	94.71	NZ_MH634990
	1,271	100	99.16	CP082876.1

The percentage query cover, sequence identity, and accession number of previously identified *C. coli* plasmids were obtained by performing a blastn of sequenced and assembled plasmids presented in column 2 of the table.

Plasmid DNA sequences from each strain were further analyzed in SnapGene (Dotmatics), and the amino acid sequence of open reading frames (ORFs) were determined to identify putative replication proteins. The 2,744 bp plasmid (pCC311) from *C. coli* 311, the 3,303 bp plasmid (pCC388) from *C. coli* 388, and the 1,271 bp plasmid (pWestmill 11) from *C. coli* Westmill 11 were chosen for further analysis due to their small size.

**Fig 3:**
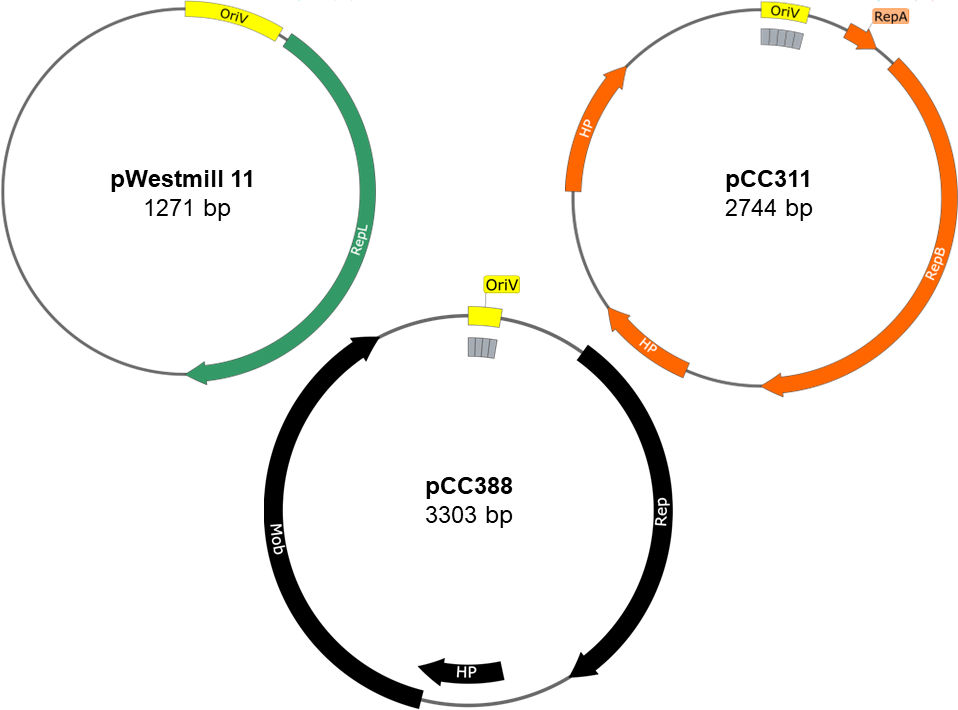
Plasmid maps of the sequenced *C. coli* Westmill 11 (green ORF), *C. coli* 311 (orange ORFs), and *C. coli* 388 (black ORFs) cryptic plasmids used to construct the shuttle vectors pJBM1, pJBM2, and pJBM3, respectively. The location and orientation of predicted replication elements (Rep), the origin of replication (OriV), and genes encoding mobilization proteins (Mob) and hypothetical proteins (HP) are indicated. Direct tandem repeats are represented by gray boxes positioned under the feature labeled OriV.

The smallest of the three plasmids, pWestmill 11 isolated from *C. coli* Westmill 11, possessed an A+T rich region lacking tandem, iteron repeats upstream of a single coding sequence corresponding to a replication/maintenance protein, RepL (WP_002799729.1) (Fig. 3). This plasmid is 36 bp smaller than a previously identified plasmid, pCC2228-1 (40), and the same size as an unnamed plasmid (CP082876). In contrast to pCC311 and pCC388, which are likely members of the theta-replicating, *repAB* iteron containing plasmids, pWestmill 11 is suspected to replicate via a rolling-circle mechanism (40).

Analysis of pCC311 from *C. coli* 311 revealed four ORFs and an A+T rich region possessing five direct tandem repeats of 22 bp (Fig. 3), characteristic of an origin of replication (iterons nt 10–110) (41). The first ORF directly downstream of this region encodes a 26 amino acid protein with 100% identity with plasmid replication protein A (ARE81508.1). The second ORF encodes a 344 amino acid protein with 98.54% identity to a RepB family plasmid replication protein (EAL2766289.1). ORF-3 and ORF-4, further downstream, encode 78 and 107 amino acid *C. coli* hypothetical proteins WP_201458541.1 and WP_002806461.1, respectively. Plasmid pCC311 is a newly characterized plasmid, different from any previously reported plasmids. Interestingly, it is smaller than other theta-replicating *C. coli* cryptic plasmids as it lacks an ORF encoding a mobilization protein typically found in other theta-replicating *C. coli* cryptic plasmids, including pCCT1 (X82079) and p3384 (42), but instead encodes two hypothetical proteins.

Analysis of pCC388 from *C. coli* 388 revealed three open reading frames and an A+T rich region possessing four direct tandem repeats of 22 bp (Fig. 3). The first ORF encodes a 345 amino acid protein with 100% identity to a replication initiation protein (ECK8531972.1). The second ORF encodes a 90 amino acid protein with 100% identity to a *C. coli* hypothetical protein (EAI7291999.1). The third ORF, immediately downstream of ORF-2, encodes a 424 amino acid protein with 99.53% identity to a *C. coli* mobilization protein (EAL7355230.1). The plasmid pCC388 is the same size as a previously identified *C. coli* cryptic plasmid, pCC2228-2 (40).

### Construction of vectors pJBM1, pJBM2, and pJBM3

**Fig 4:**
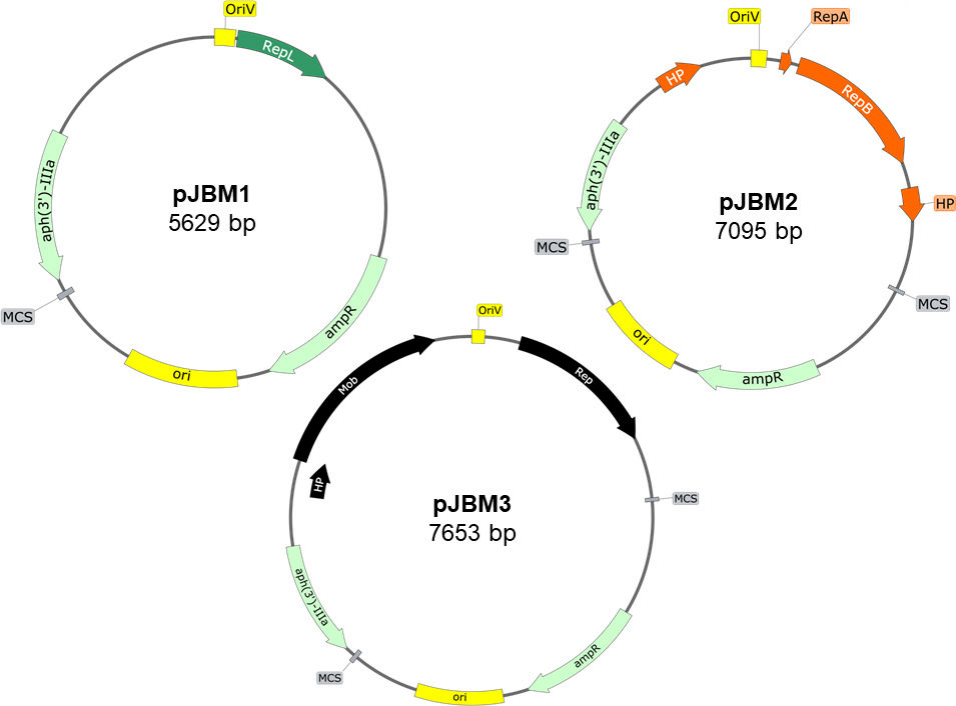
Construction of the shuttle vectors pJBM1, pJBM2, and pJBM3. Genes encoding replication proteins, hypothetical protein (HP), and mobilization protein (Mob), and the origin of replications obtained from pWestmill 11, pCC311, and pCC388 are highlighted in green, orange, and black, respectively. The remaining sequence of the vectors contains the *E. coli* origin of replication (ori), the kanamycin [*aph(3')-IIIa*], ampicillin-resistance (*ampR*) genes, and multiple cloning sites (MCS) regions from pMW2. The plasmid maps have been generated from the sequenced shuttle vectors.

To construct plasmid pJBM1 (Fig. 4), pWestmill 11 isolated from *C. coli* Westmill 11 was linearized and cloned into pMW2, a plasmid that can replicate in *E. coli* and contains a kanamycin resistance gene [*aph(3’)-IIIa*] that can be expressed in both *Campylobacter* and *E. coli* (18). To construct the *C. hepaticus-E. coli* shuttle vector pJBM2 (Fig. 4), the cryptic plasmid pCC311 carrying the predicted OriV and sequences encoding RepA, RepB, and the two hypothetical proteins was linearized and cloned into pMW2. To construct pJBM3, the cryptic plasmid pCC388 carrying the predicted OriV and sequences encoding replication initiation protein, mobilization, and the hypothetical protein was linearized and cloned into pMW2 (Fig. 4). These shuttle vectors contain multiple cloning site regions present in the pMW2 backbone (highlighted in gray) (Fig. 4).

### Transformation of *Campylobacter* with vectors pJBM1, pJBM2, and pJBM3

To investigate the functionality of the shuttle vectors, pJBM1, pJBM2, and pJBM3 were introduced into *C. hepaticus* HV10^T^ and the respective strains (*C. coli* Westmill 11, *C. coli* 311, and *C. coli* 388) from which the small cryptic plasmids included in the shuttle vectors were isolated. No recoverable transformants were obtained when *C. hepaticus* HV10^T^ was transformed with pJBM1, but *C. coli* Westmill 11 was successfully transformed with the plasmid. Shuttle vector pJBM2 replicated and was maintained in *C. hepaticus* HV10^T^ and *C. coli* 311. Shuttle vector pJBM3 replicated and was maintained in *C. hepaticus* HV10^T^ and *C. coli* 388. Shuttle vectors pJBM2 and pJBM3 were re-isolated from *C. hepaticus* HV10^T^, transformed into *E. coli* and a restriction enzyme diagnostic digest of each vector was performed (Fig. S1) to unequivocally show that each shuttle vector could be maintained and manipulated in *E. coli* and *C. hepaticus* HV10^T^.

In the above-described initial experiments, the *C. hepaticus* HV10^T^ type strain was shown to be transformable, but it was not known if this was a general characteristic common to other *C. hepaticus* strains. Therefore, the electro-competency of 11 different *C. hepaticus* strains were tested by transformation with the shuttle vector pJBM3 isolated from *E. coli* NEB 5-alpha and five with plasmid DNA isolated from *C. hepaticus* HV10^T^. Transformation efficiencies varied considerably between *C. hepaticus* strains (Table 2). The strain *C. hepaticus* HV10^T^ was the most efficient in taking up plasmid DNA compared with the other 10 strains tested (Table 2). The transformation rates obtained with plasmid DNA isolated from *C. hepaticus* and *E. coli* were similar for the five strains tested with both (Table 2). Additionally, pJBM2 and pJBM3 were introduced into *C. bilis* VicNov18^T^ to determine if the shuttle vectors developed for *C. hepaticus* could also function in *C. bilis*. A diagnostic digest of pDNA isolated from *C. bilis* VicNov18^T^ transformants and a *C. bilis*-specific PCR (Fig. S2) confirmed both shuttle vectors were functional in *C. bilis* and that *C. bilis* was the species transformed, respectively. Thus, these vectors also serve as tools to manipulate this species.

**TABLE 2:** Efficiency of electro-transformation of *C. hepaticus* and *C. bilis* strains with shuttle vector pJBM3 isolated from *E. coli* NEB 5-alpha or *C. hepaticus* HV10^T*a*^

*C. hepaticus* strain	Maximum transformation efficiency (CFU/μg) using pDNA isolated from:	
	*E. coli*	*C. hepaticus*
HV10^T^	1.5 × 10^4^	1.5 × 10^4^
19L	7.0 × 10^3^	2.8 × 10^3^
Dis-red	2.2 × 10^2^	2.8 × 10^2^
ACE4109	1.8 × 10^3^	3.5 × 10^2^
65B	3.0 × 10^2^	1.6 × 10^2^
73B	1.7 × 10^1^	1.7 × 10^1^
NSW44L	1.6 × 10^3^	Nt^*b*^
Vic oct 18	1.9 × 10^2^	Nt
Vic sept 18	3.2 × 10^2^	Nt
2T8109	7.4 × 10^3^	Nt
2204 (2)	1.2 × 10^3^	Nt
*C. bilis* VicNov18^T^	4.7 × 10^2^	Nt

^*a*^ Values represent the maximum transformation efficiencies obtained from at least two independent experiments.

^*b*^ Nt, not tested.

Transformation efficiencies of >1 × 10^4^ CFU/µg of pJBM3 into *C. hepaticus* HV10^T^ were obtained across six independent experiments. Thus, this most highly transformable strain was used for site-specific mutagenesis studies.

### *In vitro* stability of shuttle vectors

To further investigate the functionality of the shuttle vectors, the stability of pJBM2 and pJBM3 replication and maintenance in the absence of antibiotic selection was quantified. After ~50 generations (five subcultures), 100% of cross-patched colonies grew on both selective and non-selective media for both plasmids. These results indicated that the plasmids remained stable through multiple generations without antibiotic selection.

### Site-specific mutagenesis of *pglB* and complementation in *trans*

**Fig 5:**
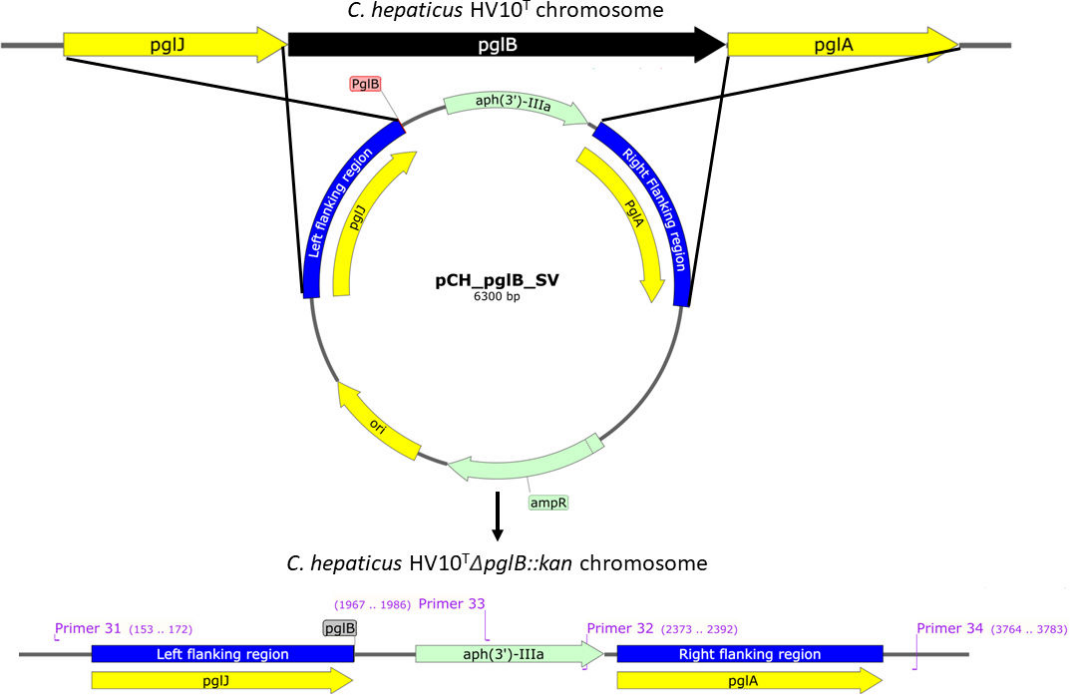
Schematic diagram of the construction of *pglB* mutants by homologous recombination. Relevant chromosomal genes are shown. The diagram illustrates the process by which the wild-type (WT) *pglB* gene from *C. hepaticus* HV10^T^ was deleted by replacement with the *aph(3’)-IIIa* on pCH_*pglB*_SV by a double crossover recombination event utilizing 1,103 and 1,120 bp homologous left and right flanking regions (blue) of *pglB,* respectively. The primers used to screen for double crossover events are provided in purple on the *C. hepaticus* HV10^T^∆*pglB::kan* chromosome.

To determine if *C. hepaticus* is amenable to mutagenesis by homologous recombination, a suicide vector, pCH_*pglB*_SV (Fig. 5), was constructed and introduced into *C. hepaticus* HV10^T^ to delete the oligosaccharyltransferase-encoding gene, *pglB*. The suicide vector was introduced into *C. hepaticus* HV10^T^ in two independent transformation reactions and produced 11 and 14 kanamycin-resistant transformants, respectively. Three and six mutants, respectively, were screened from each reaction to determine if mutants had undergone a double-crossover recombination event by PCR (Fig. S3). Any mutants produced by a single-cross over recombination event were discarded, for example, M1 and M2 (Fig. S3). The suicide vector was also introduced into *C. hepaticus* NSW44L to determine if mutagenesis could be achieved in more than one *C. hepaticus* strain. Four *C. hepaticus* NSW44L were screened as described above (Fig. S4A).

**Fig 6:**
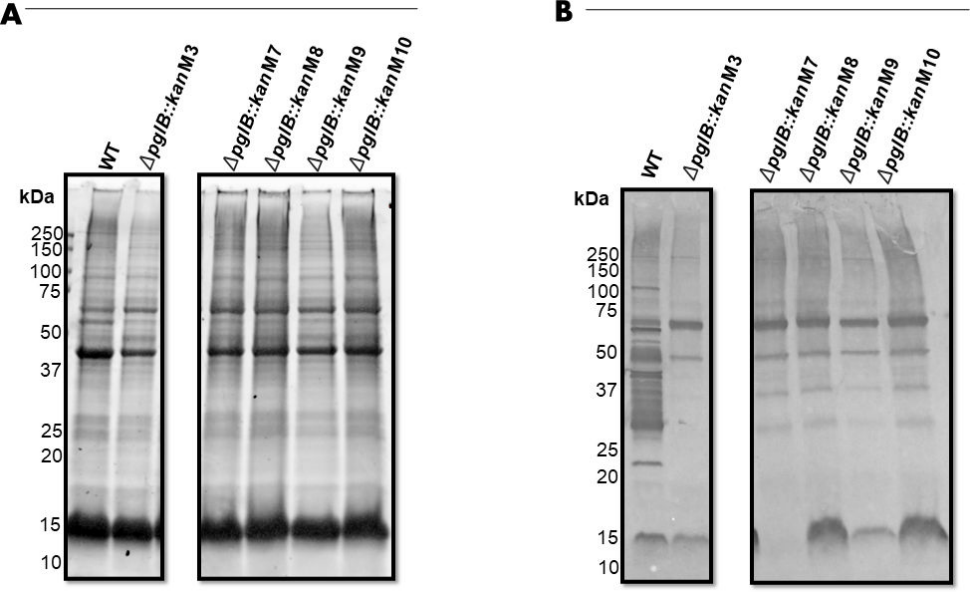
The effect of *pglB* mutagenesis on SBA binding profiles to *N-*glycans present in *C. hepaticus* HV10^T^∆*pglB::kan* mutants. Mutant 3 was independently derived from mutants 7, 8, 9, and 10. (**A**) Sodium dodecyl sulphate-polyacrylamide gel electrophoresis (SDS-PAGE) and (**B**) SBA lectin blotting of *C. hepaticus* HV10^T^ (WT) and *C. hepaticus* HV10^T^∆*pglB::kan* mutants of whole cell lysates containing 35–40 μg of protein. Whole cell lysates were separated by 8%–16% SDS-PAGE and either developed with SimplyBlue SafeStain or transferred to PVDF membranes for lectin blotting.

To confirm that deletion of the *pglB* gene resulted in the loss of *N-*glycosylated proteins, the binding of soybean agglutinin (SBA) lectin to GalNAc residues of the *N*-linked heptasaccharide glycan of multiple *C. hepaticus* HV10^T^∆*pglB::kan* mutant whole cell lysates was investigated and compared with wild-type (WT) *C. hepaticus* HV10^T^ (Fig. 6). The same phenotypic analysis was performed to compare four *C. hepaticus* NSW44L∆*pglB::kan* mutants with WT *C. hepaticus* NSW44L (Fig. S4B and C). The SBA binding profiles were significantly reduced in *C. hepaticus* HV10^T^ and *C. hepaticus* NSW44L mutant strains compared with their respective WT strains, indicating that the deletion of *pglB* had disrupted the glycosylation pathway and greatly reduced the synthesis of *N-*glycoproteins (Fig. 6B; Fig. S6C). Furthermore, these results demonstrated that mutagenesis could be performed in more than one *C. hepaticus* strain. Interestingly, *C. hepaticus* HV10^T^∆*pglB::kan* mutant 7 produced a unique banding pattern where the 15 kDa band was completely absent, whereas this phenotype was not observed in the other *pglB* mutants screened (Fig. 6B). Thus, mutant 7 was chosen for complementation work because it showed the greatest decrease in the SBA binding profile to *N*-glycans present in whole cell lysates.

Sequence analysis of *C. hepaticus* HV10^T^∆*pglB::kan* mutant 7 (JBEFTY000000000: Scaffold 0) showed that *aph*(*3’)-IIIa* had integrated by a double crossover recombination event and deleted *pglB*, as depicted in Fig. 5. Sequence analysis using the raw sequence reads (NCBI SRA SRX24788994) in Snippy identified five off-site single nucleotide polymorphisms (SNPs) in the genome. Manual genome interrogation revealed in four out of five cases that the SNPs corresponded to single base deletions in homopolymer regions. Therefore, these changes are likely to be artifacts of the sequencing process known as post-homopolymer errors (43). The fifth SNP was identified as a substitution of cystine with adenine at position 549332 in the reference genome (NZ_CP031611.1), resulting in an amino acid change from arginine to leucine in a gene encoding enoyl-ACP reductase. These enzymes are responsible for the biosynthesis of bacterial fatty acids (44), and importantly, a mutation in this gene is unlikely to affect the *C. hepaticus N-*glycosylation system and impact the phenotype.

**Fig 7:**
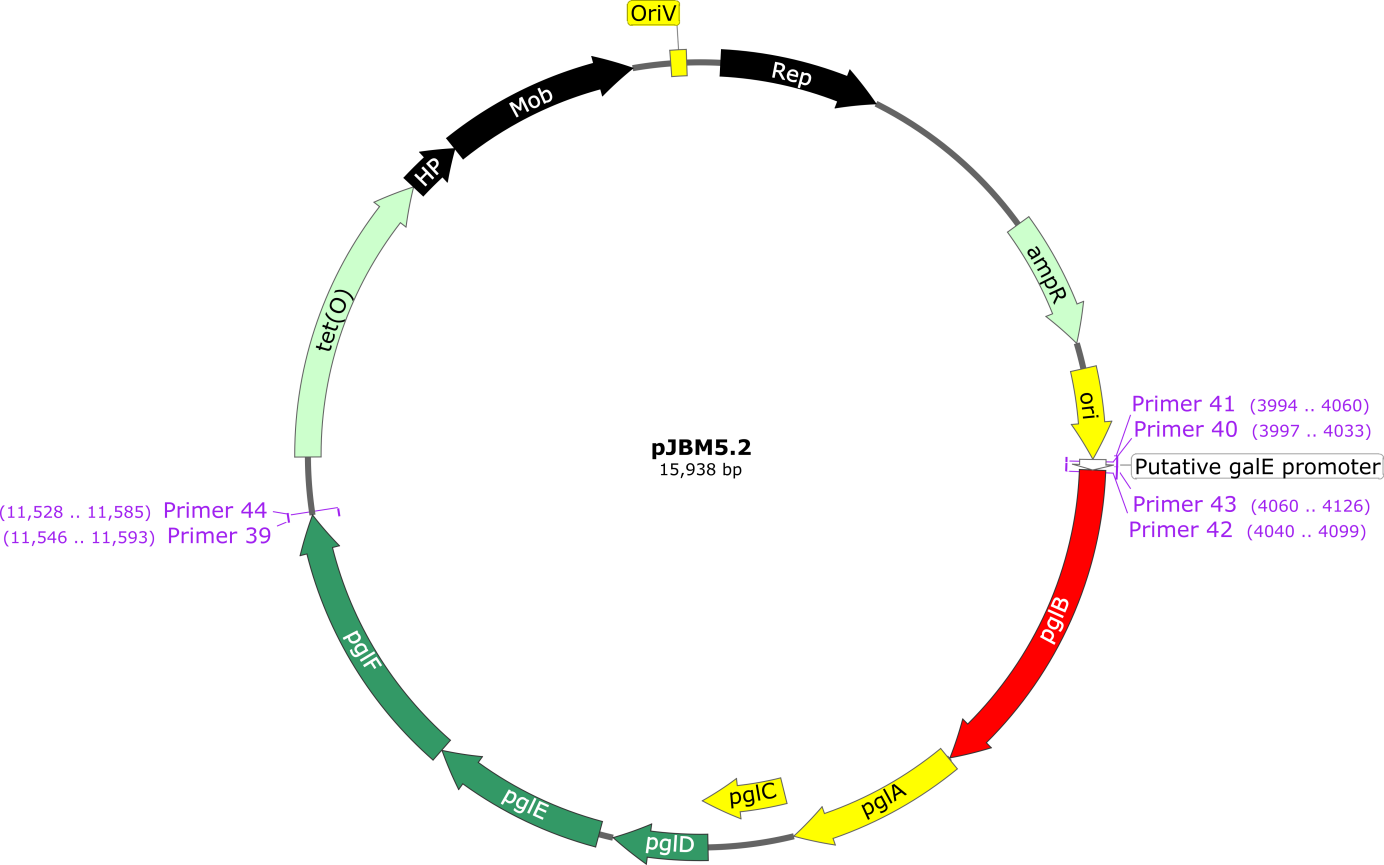
Plasmid map of shuttle vector pJBM5.2 used to complement *C. hepaticus* HV10^T^∆*pglB::kan* mutant 7. Genetic symbols represent: Rep, replication initiation protein; HP, hypothetical protein; Mob, mobilization protein; OriV, *Campylobacter* origin of replication; ampR, Ampicillin resistance gene; ori, *E.coli* origin of replication from pJBM5; *galE* putative promoter; *pglB, pglA, pglC, pglD, pglE,* and *pglF* from *C. hepaticus* HV10^T^ (Fig. 1); and *tet(O*), tetracycline resistance gene from *C. hepaticus* 84B. The position of the primers used to construct pJBM5.2 by Gibson assembly is shown in purple.

**Fig 8:**
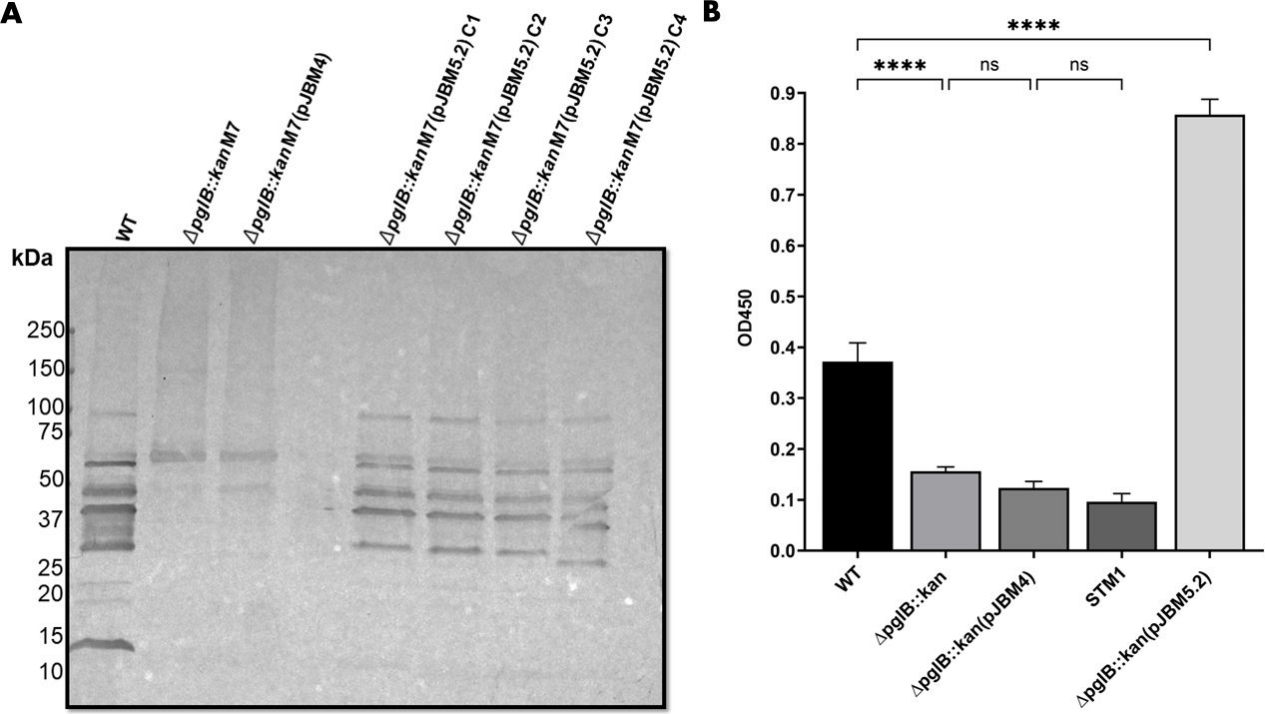
Complementation of *C. hepaticus* HV10^T^∆*pglB::kan* mutant 7 in *trans* with pJBM5.2, expressing the *pgl* locus genes including and downstream of *pglB* and excluding *pglG*. (**A**) SBA lectin blot binding profiles to *N-*glycans present in *C. hepaticus* HV10^T^, *C. hepaticus* HV10^T^∆*pglB::kan* mutant 7, *C. hepaticus* HV10^T^∆*pglB::kan* mutant 7(pJBM4) (empty vector control), and two clones from separate isogenic complements (clones 1 and 2 were derived independently of clones 3 and 4) of *C. hepaticus* HV10^T^∆*pglB::kan* mutant 7 clones expressing functional *pgl* genes encoded on pJBM5.2. Whole cell lysates contained 27 µg of protein and were separated by 4%–20% SDS-PAGE and either developed with SimplyBlue SafeStain or transferred to PVDF membranes for lectin blotting. The corresponding SDS-PAGE gel to demonstrate equal loading of proteins across samples is provided in Fig. S8. (**B**) Quantitative analysis of SBA binding to GalNAc present in whole cell lysates. ELISA results represent SBA binding to whole cell lysates of *C. hepaticus* HV10^T^, *C. hepaticus* HV10^T^∆*pglB::kan* mutant 7, *C. hepaticus* HV10^T^∆*pglB::kan* mutant 7(pJBM4), *C. hepaticus* HV10^T^∆*pglB::kan* mutant 7(pJBM5.2), and *Salmonella* Typhimurium 82/6915 ∆*aroA* (STM1) (negative control for *N*-glycosylation) measured at OD_450_. Gray bars represent the standard deviation of *n* = 4 technical replicates for each strain. Statistical differences between groups were analyzed using a one-way ANOVA with Tukey’s post hoc analysis and are indicated as: ns, no statistically significant difference (*P*-value > 0.05); and **** indicate statistically significant differences with *P*-values < 0.0001. For all Tukey’s multiple comparisons, see Table S1.

To complement the *C. hepaticus* HV10^T^∆*pglB::kan* mutant 7, pJBM3 was used to construct a series of vectors to restore the *N-*glycosylation of proteins in *C. hepaticus* HV10^T^∆*pglB::kan* mutant 7. First, pJBM4 (Fig. S5) was constructed to serve as an empty vector control for using pJBM3 as a backbone for complementation of *C. hepaticus* HV10^T^∆*pglB::kan* mutant 7. This was achieved by replacing *aph*(*3’)-IIIa* with the *tet*(O) gene obtained from the pCJDM210L-like plasmid isolated from *C. hepaticus* 84B. Next, pJBM5.1, which carried a single copy of *pglB* under the control of the putative *galE* promoter (Fig. S6) was constructed and introduced into *C. hepaticus* HV10^T^∆*pglB::kan* mutant 7 but did not restore the *N-*glycosylation of proteins in this strain (Fig. S7). This was likely due to polar effects caused by the mutagenesis process impacting the transcription and expression of genes downstream of *pglB*. Attempts to complement another mutant strain (M3) with a single copy of *pglB* were unsuccessful (data not shown), suggesting polar effects in that mutant too. Thus, pJBM5.2, expressing the *pgl* locus genes including and downstream of *pglB* (Fig. 7), was designed and introduced into *C. hepaticus* HV10^T^∆*pglB::kan* mutant 7. Complementation of the mutant strain was assessed based on the restoration of SBA binding to *N*-glycans present in whole cell lysates from complemented mutants compared with WT *C. hepaticus* HV10^T^ (Fig. 8) using western blotting and an enzyme linked immunosorbent assay.

Introduction of pJBM5.2 into *C. hepaticus* HV10^T^∆*pglB::kan* mutant 7 partially complemented SBA binding to *N-*glycans (Fig. 8) in four clones across two independently derived isogenic complements. Six distinct bands between 25 and 100 kDa were restored to WT levels in the complemented clones transformed with pJBM5.2 (Fig. 8) that were absent in the mutant strain and the mutant strain transformed with the empty vector control, pJBM4. Notably, a dominant band at 15 kDa present in *C. hepaticus* HV10^T^, suspected to correspond to lipooligosaccharide (LOS) (38), could not be restored in the complemented mutant strain.

The results of the ELISA measurement of the SBA binding to the heptasaccharide glycan support the results observed in the lectin blot (Fig. 8A). SBA binding to the heptasaccharide present in whole cell lysates of *C. hepaticus* HV10^T^∆*pglB::kan* mutant 7 and *C. hepaticus* HV10^T^∆*pglB::kan* mutant 7(pJBM4) was greatly reduced to levels comparable with STM1 (lacks *N-*glycosylation) compared with WT and the complemented mutant strain (Fig. 8B). Notably, the levels of SBA binding were significantly higher (approximately two times) in the complemented mutant strain compared with WT.

## DISCUSSION

This study reports the successful genetic manipulation of *C. hepaticus* by site-specific mutagenesis via allelic exchange. The construction of *E. coli-C. hepaticus* shuttle vector plasmids enabled the development of a DNA electro-transformation method, which was effective for introducing plasmid DNA into *C. hepaticus* and *C. bilis* and was of sufficient efficiency to recover double recombination events (knockout mutants) after transformation with a non-replicating suicide vector.

A series of *E. coli-C. hepaticus* shuttle vectors have been developed utilizing the replication machinery from two small cryptic plasmids from two different *C. coli* isolates. The cryptic plasmids used in the current study are theta-replicating, iteron-containing plasmids that belong to the same incompatibility group as previously described *Campylobacter* shuttle vectors (18, 20, 30). The cryptic plasmid pCC311 is at least 456 bp smaller than other previously characterized *C. coli* theta-replicating cryptic plasmids. The size of shuttle vectors pJBM2 and pJBM3 could be further reduced by excision of the *ampR* gene, which is non-functional in *Campylobacter*. This could reduce the size of the shuttle vector by approximately 1,000 bp. Another putative *E. coli-C. hepaticus* shuttle vector, pJBM1, utilizing a 1,271 bp cryptic plasmid previously characterized and suspected to replicate by a rolling-circle mechanism (40), was constructed but could not replicate in *C. hepaticus*. This is likely due to the *repL* gene being insufficient for plasmid replication and maintenance in *C. hepaticus* HV10^T^.

The shuttle vector plasmids pJBM2 and pJBM3 were functional in *C. coli* 311 and *C. coli* 388, respectively, and both in *C. bilis* VicNov18^T^*,* demonstrating their versatility and use for gene expression in other *Campylobacter* species. The plasmids were also stably maintained without antibiotic selection in *C. hepaticus* HV10^T^. This was not surprising as Miller and others (45) previously demonstrated >95% plasmid stability of *C. jejuni* shuttle vectors developed using cryptic plasmid elements as replication machinery after a similar number of generations without antibiotics. The number of generations in the current study (50) was chosen due to the need to grow *C. hepaticus* on solid-growth medium plates rather than in liquid culture. Assessment of plasmid stability could not be achieved using liquid culture due to the fastidious nature of *C. hepaticus*. The stability of pJBM2 and pJBM3 highlights their usefulness as tools for the genetic complementation of *C. hepaticus* HV10^T^ mutant strains for future *in vivo* studies.

Transformation efficiencies varied between *C. hepaticus* strains, which was expected as this has been observed for *C. jejuni,* where some strains are highly electrocompetent and others cannot be transformed with plasmid DNA by electroporation (16, 17, 28, 30). To our surprise, transformation efficiencies of *C*. *hepaticus* transformed with plasmid DNA isolated from *E. coli* did not differ significantly from *C*. *hepaticus* transformed with plasmid DNA isolated from itself. The higher number of transformants observed in *C. hepaticus* strains transformed with plasmid DNA isolated from *E. coli* compared with plasmid DNA isolated from *C. hepaticus* may be due to higher quality plasmid DNA preparations from *E. coli*. Previous studies have shown that the source of plasmid DNA has a significant impact on the efficiency of transformation whereby most *C. jejuni* strains cannot be transformed with plasmid DNA isolated from *E. coli,* or efficiencies of transformation are reduced by four orders of magnitude due to a DNA restriction/modification system responsible for restricting heterologous DNA uptake (17, 28). Holt and others showed that *C. jejuni* NCTC 11168 possesses a gene, *cj1051c* (*cjeI*)*,* encoding a type II restriction and methyl transferase enzyme, which significantly decreases transformation efficiencies with plasmid DNA (46). Furthermore, a mutation in *cjeI* permitted transformation with plasmids isolated from an *E. coli* host (46). In contrast, the current study suggests no DNA restriction barrier between *C. hepaticus* and *E. coli*. A blastp search of *cjeI* restriction-modification enzyme against *C. hepaticus* HV10^T^ produced no significant sequence similarity, suggesting this enzyme is absent in *C. hepaticus* HV10^T^, which may modestly explain this phenomenon.

Next, the feasibility of gene deletion and replacement via homologous recombination for the mutagenesis of *C. hepaticus* HV10^T^ and *C. hepaticus* NSW44L was demonstrated by producing deletion mutants of *pglB*. It was recently shown that *C. hepaticus* possesses an *N-*glycosylation system that modifies proteins with a heptasaccharide glycan (38). Most of the glycosylated proteins identified were highly conserved glycoproteins, also present in *C. jejuni*, that play crucial roles in host colonization (47, 48). Some glycoproteins were found to be unique to *C. hepaticus*, which may contribute to its ability to cause SLD. Mutations in *C. jejuni pgl* genes have profound adverse effects on proteome stability, growth, cellular function, its virulence in humans, and host colonization (47–49). Thus, *pglB,* the oligosaccharyltransferase that is essential for the *N*-glycosylation of proteins in *C. jejuni* (31, 50), was chosen as a target for gene deletion in *C. hepaticus* HV10^T^.

The *pglB* gene was successfully mutated in *C. hepaticus* HV10^T^ and *C. hepaticus* NSW44L via allelic exchange using a suicide vector lacking a contra-selective marker. The strain NSW44L was chosen in addition to the *C. hepaticus* type strain (HV10^T^) as this strain was previously shown to be significantly more virulent compared with the type strain and another *C. hepaticus* strain (51). The ability to perform mutagenesis in a highly virulent strain is desirable so that the phenotypic effects of knockouts of various genes encoding potential virulence factors can be assessed in animal studies. Homologous DNA regions of approximately 1,000 bp were used in the suicide vector developed to induce allelic exchange, as increasing the average length of homologous DNA regions on suicide vectors has been shown to improve recombination efficiency in *C. jejuni* (17). Phenotypic analysis of SBA binding profiles to whole cell lysates of *C. hepaticus pglB* mutants using a lectin, SBA, which binds to the terminal GalNac residues of the heptasaccharide present on glycoproteins (38, 52), showed that the *pglB* gene is critical for generating *N-*glycoproteins in *C. hepaticus* HV10^T^ and *C. hepaticus* NSW44L*.* The SBA reactive bands present in the lectin blots of whole cell lysates of the mutant 7 strain *(*Fig. 6 and 8) are likely due to non-specific binding of the lectin. This is further supported by the quantitative measurement of SBA binding to the heptasaccharide, which demonstrated that *S*. Typhimurium STM1 levels were comparable with the empty vector control mutant strain. *S*. Typhimurium lacks an *N*-glycosylation system, indicating that the signals seen are likely due to non-specific reactions and, more importantly, suggests that deletion of *pglB* completely abolished the production of *N*-glycoproteins in *C. hepaticus* HV10^T^. Additional unexpected reactivity of SBA lectin to whole cell extracts of other *Campylobacter* species has previously been attributed to non-specific interactions (32). These data confirm the essential role of *pglB* in the glycosylation reactions as the suspected oligosaccharyltransferase.

To demonstrate the utility of the newly developed shuttle vectors, the *C. hepaticus* HV10^T^∆*pglB::kan* mutant 7 was complemented in *trans* to restore glycosylation activity. Initial attempts to complement by expressing only the native *pglB* gene under the control of the *C. hepaticus* HV10^T^ *galE* putative promoter on pJBM3 were unsuccessful. It was hypothesized that this failure to complement was likely due to polar effects caused by the deletion of *pglB*. A previous study showed that deletion of *pglB* in *C. jejuni* resulted in decreased abundance of other essential enzymes encoded by *pgl* genes downstream of *pglB,* including PglF, which is responsible for the biosynthesis of bacillosamine, a rate-limiting step for the *N-*glycosylation pathway (36). The abundance of enzymes encoded by genes upstream of *pglB* was unaffected (36), supporting the rationale for the construction of a complementation vector containing *pglB* and the *pgl* genes downstream of *pglB*. The *pglB* gene and all genes downstream of *pglB* in the *pgl* locus, excluding *pglG*, were cloned into pJBM5 under the control of the *galE* promoter to counteract any of these polar effects. The gene, *pglG,* was excluded from the vector to reduce the size of the complementation vector as a previous study had shown that mutagenesis of *pglG* had no impact on SBA reactivity to *C. jejuni* whole cell lysates and likely plays no role in glycan biosynthesis (39). The complementation vector, pJBM5.2, partially restored the production of *N-*glycosylated proteins in the *pglB* mutant strain. Interestingly, quantitative analysis of SBA binding to the heptasaccharide glycan in whole-cell lysates revealed that the relative abundance of the heptasaccharide in the complemented strain was significantly greater than WT levels. It is hypothesized that this was due to the presence of multiple DNA copies (in *trans* on the multi-copy number plasmid) of the *galE* promoter and all genes downstream of *pglB*, resulting in higher than WT levels of gene expression. For example, the enzymes involved in the early stages of heptasaccharide synthesis, including *PglA,* responsible for adding the first GalNAc residue of the heptasaccharide, may be expressed at amounts greater than WT, thus elevating the levels of heptasaccharide produced, which may not be bound to protein but could be present as additional free oligosaccharide.

Notably, the SBA lectin blot phenotype could not be completely reverted to WT levels in the complemented strain. A dominant band present on the SBA lectin blot at 15 kDa for the WT strain has previously been shown to be non-proteinaceous and likely corresponds to *C. hepaticus* HV10^T^ LOS (38), which could not be complemented in the *pglB* mutant strain. The reasons why this band was impacted during mutagenesis and why it could not be complemented in this particular mutant strain remain unknown. A previous study demonstrated *pglB* mutagenesis in *C. jejuni* results in a significant decrease in isoprenyl transferase, UppS, a crucial enzyme involved in the synthesis of undecaprenyl diphosphate, which is an essential lipid carrier that plays key roles in the biosynthesis of LOS in *C. jejuni* (35). This would explain the loss of SBA binding to the 15 kDa band in the *C. hepaticus* HV10^T^ ∆*pglB::kan* mutant 7 strain. Therefore, the expression of UppS to increase the abundance of Und-P may be needed to restore the synthesis of LOS and SBA binding to the 15 kDa band. The disruption of LOS production may have implications on cell physiology and morphology, which warrants further investigation.

In summary, the newly developed capability to produce targeted mutations in *C. hepaticus* has provided the first direct evidence of *pglB* involvement in the *N-*glycosylation of *C. hepaticus* HV10^T^ proteins. To confirm that *N*-glycosylation in *C. hepaticus* HV10^T^ is an essential pathogenic determinant of SLD, future research should utilize the tools, methods, *pglB* mutant strains, and complemented strains developed in this study to investigate if *C. hepaticus* lacking *pglB* can cause SLD in the poultry model of disease. The tools developed could also be used to improve our understanding of the direct and indirect effects of disrupting the *N*-glycosylation system on proteome stability and other critical cellular pathways linked to virulence of *C. hepaticus* and other *Campylobacter* species*.* The new genetic manipulation technologies developed in this study will aid in the study of *C. hepaticus* virulence and SLD pathogenesis and provide a pathway for the development of effective intervention strategies against SLD that can reduce the economic and animal welfare impact of SLD.

## MATERIALS AND METHODS

### Bacterial strains, media, and growth conditions

The bacterial strains and plasmids used in this study are described in Table 3. The *Campylobacter* strains were grown on Brucella agar (Amyl Media) with 5% horse blood (HBA) plates and incubated at 42°C under microaerophilic conditions (85% N_2_, 10% CO_2_, and 5% O_2_) using CampyGen gas packs (Oxoid) for 24–72 h. *E. coli* NEB 5-alpha (New England Biolabs) was used for cloning and plasmid propagation. *E. coli* and *S*. Typhimurium were grown in Luria-Bertani (LB) (BD Difco) broth or medium at 37°C overnight. When appropriate, HBA and LB were supplemented with antibiotics; kanamycin (30 µg/mL for *Campylobacter* and 50 µg/mL for *E. coli*), ampicillin (100 µg/mL) for *E. coli*, and tetracycline (10 µg/mL) for *E. coli* and *Campylobacter*.

**TABLE 3:** Bacterial strains and plasmids used in this study

Strain or plasmid	Description	Source or reference
Bacterial strains		
*C. hepaticus* HV10^T^ (NCTC 13823)	Wild-type reference strain	(6)
*C. hepaticus* 84B	Wild-type strain with a 44.9 kb horizontally acquired tetracycline resistance plasmid	(14)
*C. hepaticus* 19L	Wild-type strain	(14)
*C. hepaticus* NSW44L	Wild-type strain	(14)
*C. hepaticus* DISRED	Wild-type strain	(14)
*C. hepaticus* ACE4109	Wild-type strain	RMIT collection
*C. hepaticus* 65B	Wild-type strain	RMIT collection
*C. hepaticus* 73B	Wild-type strain	RMIT collection
*C. hepaticus* VicOct 18	Wild-type strain	RMIT collection
*C. hepaticus* VicSept 18	Wild-type strain	RMIT collection
*C. hepaticus* 2T8109	Wild-type strain	RMIT collection
*C. hepaticus* 220211	Wild-type strain	RMIT collection
*C. bilis* VicNov18^T^	Wild type reference strain	(11)
*C. coli* Westmill 11	Strain with cryptic plasmid with 100% query cover and 99.16% identity with CP082876.1	RMIT collection
*C. coli* 311	Strain with cryptic plasmid with 75% query cover and 91.11% identity with pCCT1 X82079.1	RMIT collection
*C. coli* 388	Strain with cryptic plasmid with 100% query cover and 97.55% identity with pR19.0802_3.3k CP076512.1	Coloe (53) RMIT collection
*C. hepaticus* HV10^T^ ∆*pglB::kan*	HV10^T^ with deletion in oligosaccharyltransferase gene, *pglB*; A2J15_RS01350 by replacement with a kanamycin resistance gene	This study
*C. hepaticus* NSW44L ∆*pglB::kan*	NSW44L with deletion in oligosaccharyltransferase gene, *pglB*; by replacement with a kanamycin resistance gene	This study
*C. hepaticus* HV10^T^ ∆*pglB::kan* M7(pJBM5.1)	HV10^T^ *pglB* mutant encoding a wild-type copy of *pglB,* expressed on pJBM5.1	This study
*C. hepaticus* HV10^T^ ∆*pglB::kan* M7(pJBM5.2)	HV10^T^ *pglB* mutant complemented with wild-type copies of *pgl* genes *pglB, A, C, D, E,* and F expressed on pJBM5.2	This study
*E. coli* NEB 5-alpha	A derivative of *E. coli* DH5α used for cloning and plasmid propagation. *fhuA2*∆*(argF-lacZ)U169 phoA glnV44* Φ*80*∆*(lacZ)M15 gyrA96 recA1 relA1 endA1 thi-1 hsdR17*	New England Biolabs
*Salmonella* Typhimurium 82/6915 (STM1)	An *aroA* mutant of *S*. Typhimurium strain 82/6915	Bioproperties Pty Ltd Collection
*E. coli* JM109	An *E. coli* K strain used for cloning and plasmid propagation*. endA*, *recA1 gyrA96 thi, hsdR17* (r_k_^–^, m_k_^+^) *relA1 supE44* ∆(*lac-proAB*) [F′ *traD36 proAB laq*I^q^Z∆M15]	Promega
Plasmids		
pMW2	pBluescript KS containing a Km^R^ cassette	(54)
pCJDM210L like	Vector with tetracycline-resistant gene *tet*(O) isolated from *C. hepaticus* 84B	(14)
pWestmill 11	Cryptic plasmid isolated from *C. coli* Westmill 11 used for the construction of pJBM1	This study
pCC311	Cryptic plasmid isolated from *C. coli* 311 used for the construction of pJBM2	This study
pCC388	Cryptic plasmid isolated from *C. coli* 388 used for the construction of pJBM3	This study
pJBM1	A 5,629 bp vector; Kan^R^	This study
pJBM2	A 7,095 bp *E. coli-C. hepaticus* shuttle vector; Kan^R^	This study
pJBM3	A 7,653 bp *E. coli-C. hepaticus* shuttle vector; Kan^R^	This study
pJBM4	A 8,830 bp *E. coli-C. hepaticus* shuttle vector; Tet^R^	This study
pCH_*pglB*_SV	pMW2 with *C. hepaticus pglB* flanking regions and *aph(3')-IIIa*. A suicide vector to delete *pglB*	This study
pJBM5	A 10,685 bp vector with *pglB* and *tet*(O) cloned into pJBM3	This study
pJBM5.1	A 10,548 bp vector with *galE* putative promoter cloned into pJBM5 to complement ∆*pglB::kan* mutant 7	This study
pJBM5.2	A 15,938 bp vector with *galE* putative promoter*, pglB, pglA, pglC, pglD, pglE, pglF* cloned into pJBM5 to complement ∆*pglB::kan* mutant 7	This study

### Plasmid and genomic DNA isolation and preparation

Plasmid DNA was isolated from *C. coli* and *C. hepaticus* strains grown on HBA plates for 24 h and then harvested by flooding plates with 2 mL Brucella broth and gently scraping cells off with a plastic spreader bar. The cells were transferred to a tube, centrifuged, and the cell pellet collected for DNA extraction. Plasmid DNA isolated from *E. coli* was obtained from 5 mL overnight cultures. All plasmid preparations were performed using Monarch Plasmid Miniprep Kit (NEB) according to manufacturer’s instructions. Genomic DNA was isolated from *C. hepaticus* HV10^T^ WT and mutant strains, grown for 24 h using a Monarch Genomic DNA Purification Kit (NEB) according to the manufacturer’s instructions. Plasmid and genomic DNA was quantified using either a Qubit 1× dsDNA Quantitation, high sensitivity, or Broad range assay kit (Invitrogen). The quality of plasmid and genomic DNA was determined by agarose gel electrophoresis and analyzing A260/280 absorbance ratios using a NanoDrop spectrophotometer (Thermo Fisher).

### Sequence analysis of *C. coli* cryptic plasmids and *C. hepaticus pglB* mutant strains

Plasmid DNA and genomic DNA sequencing libraries were prepared using the Illumina DNA Library Prep (M) Tagmentation (24 samples, IPB) kit according to the manufacturer’s protocol. Libraries were sequenced on a MiSeq machine (Illumina) using v3 reagents with 2 × 300 bp paired end reads according to the manufacturer’s instructions. Sequencing reads were analyzed by FastQC version 0.11.9 (55), and any reads with quality scores of <Q30 were trimmed using Trimmomatic version 0.36.6 (56).

Plasmidomes were assembled using plasmidSPAdes Galaxy Version 3.15.4+galaxy2 (57), or untreated reads were assembled with Unicycler v0.5.0 (58). All software used for analysis was accessed via Galaxy Australia (59). Assembled plasmid sequences were first used as queries in blastn (60) searches to identify significant sequence similarities to other previously reported *Campylobacter* plasmids. Sequences were uploaded to SnapGene version 7.2.(Dotmatics) and manually annotated. The amino acid sequence of ORFs present on the plasmids was determined by blastx to identify plasmid replicons. Plasmid sequences were manually analyzed to identify any A+T rich regions containing direct tandem repeats characteristic of a replication origin.

The *C. hepaticus* HV10^T^∆*pglB::kan* mutant genomes were assembled using the A5-MiSeq pipeline version 20150522 (61). Snippy version 4.6.0, accessed via Galaxy Australia was used to identify SNPs in the paired-end reads of mutant strains aligned against the reference genome (GenBank accession no. CP031611.1). Any SNPs identified by Snippy were manually interrogated by blasting assembled mutant strain contigs against *C. hepaticus* HV10^T^ reference genome to confirm the presence of any SNPs.

### Construction of *E. coli–C. hepaticus* shuttle vectors

Vector pJBM1; a 1,413 bp *Xho*I fragment was amplified, using primers 1 and primer 2, from pWestmill 11. The PCR product was cloned into the *Xho*I site of pMW2, which had been linearized with *Xho*I (NEB) and treated with Quick CIP (NEB). The vector was introduced into *E. coli* JM109 (Promega) by heat shock according to manufacturer’s instructions. Shuttle vectors pJBM2, pJBM3, and pJBM4 were constructed by Gibson assembly (62). Gibson assembly primers (Table S2) were designed using SnapGene (Dotmatics). Briefly, to construct shuttle vectors pJBM2 and pJBM3, pMW2 and the cryptic plasmids pCC311 and pCC388, containing the origin of replication and different replicons, were linearized by PCR using Q5 High-Fidelity DNA polymerase (NEB) and the respective primer pairs (Table S2). All PCR reactions for cloning were analyzed by agarose gel (0.8%–1%) electrophoresis and either cleaned up using a Monarch PCR & DNA Cleanup Kit (NEB) or the amplicons were extracted from the gel using a Monarch DNA Gel Extraction Kit (NEB) and quantified using a Qubit 1× dsDNA Quantitation, high sensitivity assay kit (Invitrogen). Vector fragments were then assembled using GeneArt Gibson Assembly Cloning HiFi Master Mix (Invitrogen) as per the manufacturer’s instructions.

The assembled vectors were transformed into *E. coli*. Transformants were selected on LB kan plates; pJBM1, pJBM2, and pJBM3 were reisolated from clones, and diagnostic digests were performed (Fig. S1). To construct the shuttle vector pJBM4, pJBM3 was used as a backbone and linearized to remove the kanamycin gene [*aph(3’)-IIIa*]*,* which was replaced with *tet*(O) and its putative promoter, obtained from pCJDM210L like plasmid, native to *C. hepaticus* 84B. These amplicons were obtained by PCR using Q5 High-Fidelity DNA polymerase (NEB) and the respective primer pairs (Table S2.) and analyzed as described above. The assembled vector was transformed into *E. coli* as above and transformants selected on LB tet plates. Shuttle vectors pJBM2, pJBM3, and pJBM4 were reisolated from *E. coli* cells and sequenced as described above.

### Transformation of *Campylobacter hepaticus*

Electrocompetent cells were prepared as described in (28) with some modifications. Briefly, frozen stocks of *C. hepaticus* strains were grown for 72 h at 42°C under microaerophilic conditions. Cells were subcultured onto two separate HBA plates and grown for an additional 20–24 h. Plates were chilled in the fridge for 15 min, and each plate was flooded with 2 mL of ice-cold wash buffer (272 mM sucrose, 15% glycerol), and cells were harvested by gentle scraping. Cells were pelleted at 5,000 × *g* for 10 min at 4°C and resuspended in 1 mL of ice-cold wash buffer. Cells were pelleted and washed twice in ice-cold wash buffer. Each cell pellet was resuspended in 125 µL of wash buffer, resulting in a cell density of approximately 10^10^–10^11^ CFU/mL as determined by plating serial dilutions of cells. Electroporation cuvettes (2 mm) were cooled on ice. Plasmid DNA was added to 50 µL of electrocompetent cells and incubated for 10 min on ice. Samples were electroporated using the parameters 2.5 kV, 200 ohms, 25 uF producing a time constant of 4.6–4.8 ms. Cuvettes were immediately flushed with 100 µL of Super Optimal broth with Catabolite repression, 2% tryptone, 0.5% yeast extract, 10 mM NaCl, 2.5 mM KCl, 10 mM MgCl_2_, 10 mM MgSO_4_, and 20 mM glucose (S.O.C) medium (Invitrogen) and recovered on non-selective HBA plates for 6 h at 42°C under microaerophilic conditions. Cells were harvested from recovery plates by flooding with 1 mL Brucella broth and pelleted at 5,000 × *g* for 3 min. Cells were resuspended in 100 µL of Brucella broth and spread plated onto HBA supplemented with the appropriate antibiotic. Cells were grown for 4–7 days under microaerophilic conditions. All other *Campylobacter* species used in this study were transformed following the same procedure.

### *In vitro* plasmid stability assay

To measure the stability of the shuttle vectors, *C. hepaticus* HV10^T^ transformed with pJBM2 and pJBM3 were resuscitated, streaked, and grown on HBA kan plates under microaerophilic conditions at 42°C for 72 h to ensure plasmid carriage. Individual colonies were picked and resuspended in 200 µL of Brucella broth, and the OD_600_ was measured using a UV-spectrophotometer (Eppendorf). After incubation, 100 µL of these bacterial suspensions were spread-plated onto non-selective HBA plates and grown under microaerophilic conditions at 42°C for 48 h. Cells were harvested using a 10 µL loop, resuspended in 200 µL of Brucella broth, and the OD_600_ was measured. The change in OD_600_ was used to determine the number of generations/doublings the cultures had undergone. After 50 generations, serial dilutions of cultures were performed in triplicates, and 100 µL of the appropriate dilutions were plated and grown for 72 h on non-selective HBA plates as described above. A total of 100 colonies from each replicate were cross-patched onto selective and non-selective HBA plates and grown for 24 h to determine the percentage of kanamycin-resistant CFU among the total number of CFU. The assay was performed in three independent biological replicates using technical triplicates.

### Site-specific mutagenesis and complementation of *pglB* in *C. hepaticus* HV10^T^

Construction of *C. hepaticus* HV10^T^∆*pglB::kan* mutants was achieved by allelic exchange using the suicide vector, pCH_*pglB_*SV, constructed using a four-fragment Gibson assembly. The flanking regions on the left (1,143 bp) and right side (1,160 bp) of the *pglB* gene were amplified using Q5 High-Fidelity DNA polymerase and the oligonucleotide primer pairs 17/18 and 21/22, respectively. These regions were cloned on their respective sides of the *aph(3’)-IIIa* gene amplified using primer pair 19/20 and assembled into the linearized pMW2 backbone using primer pair 15/16. The assembled vector was electroporated into *E. coli* and colonies selected on LB kan plates. Plasmid DNA was reisolated from *E. coli* transformants, and large quantities of the plasmid were prepared using a Qiagen Plasmid Midi Kit and 3.7 µg of the final suicide vector was electrotransformed into *C. hepaticus* HV10^T^ and *C. hepaticus* NSW44L as described above.

Transformants were screened for Integration of the *aph(3’)-IIIa* gene into the *pglB* locus by double homologous recombination using *pgl* locus and kan-specific primer pairs 31/32 and 33/34 (Table S2) and sequence analysis. The *pglB* complementation vectors pJBM5.1 (Fig. S6) and pJBM5.2 (Fig. 7) were constructed using a two and three-fragment Gibson assembly, respectively. To construct pJBM5.1 the putative *galE* promoter was cloned into the shuttle vector pJBM5. The *galE* promoter was predicted using BPROM (63) and amplified using primer pair primer 37/38. The shuttle vector pJBM5 was linearized using the primer pair 35/36 and the fragments assembled. To construct pJBM5.2, the putative *galE* promoter, and a large DNA fragment carrying *pglB, pglA, pglC, pglD, pglE*, and *pglF* were cloned into the shuttle vector pJBM5. The putative *galE* promoter was amplified using primer pair 41/42. The *pgl* genes were amplified using primer pair 43/44. The shuttle vector pJBM5 was linearized using the primer pair primer 39/40, and the fragments were assembled. The assembled vectors were transformed into *E. coli* as per manufacturer’s instructions and colonies selected on LB tet plates. The complementation vectors pJBM5.1 and pJBM5.2 were introduced into *C. hepaticus* HV10^T^∆*pglB::kan* mutant 7 strain by electroporation, and tetracycline-resistant transformants were selected.

### Whole cell lysates for ELISA and western blotting

Freshly grown cells grown under microaerophilic conditions at 42°C for 24–48 h were harvested and washed twice in phosphate buffered saline (PBS). Cells were pelleted by centrifugation at 5,000 × *g* for 5 min, and cell pellets were resuspended in 97.5 µL PBS, 2.5 µL lysozyme (100 mg/mL), and 100 µL of tissue lysis buffer (Monarch DNA Gel Extraction Kit, NEB). Samples were vortexed and heated at 37°C for 5 min. Samples underwent centrifugation at 5,000 × *g* for 5 min to remove any insoluble material, and the supernatants were collected. Protein quantification was determined using a Qubit Protein Assay kit: Q33211 (Invitrogen). Protein samples were stored at −20°C. Lectin blotting was performed using SBA (Vector Laboratories) as described in (38).

### Quantification of SBA binding to *N-*glycans in *pglB* mutant strains

A 96-well plate ELISA assay was developed to quantify and compare the abundance of heptasaccharide glycan in *C. hepaticus* HV10^T^ WT with *C. hepaticus* HV10^T^*pglB* mutant strain and the *C. hepaticus* HV10^T^ complemented mutant strain. A 96-well High Bind Microplate (Corning) was coated with 50 µL of whole cell lysates containing 200 µg/mL% protein diluted in 0.5 M carbonate buffer from *C. hepaticus* HV10^T^, *C. hepaticus* HV10^T^∆*pglB::kan* M7, *C. hepaticus* HV10^T^∆*pglB::kan* M7(pJBM4), *C. hepaticus* HV10^T^∆*pglB::kan* M7(pJBM5.2), STM1 (negative control), and 0.5 M carbonate buffer as a blank control. The plate was incubated at RT for 1.5 h. Unbound protein was removed, and the plate was blocked at 4°C overnight with 200 µL of 5% BSA. After discarding the blocking solution, the plate was probed with 100 µL of SBA (2 µg/mL) diluted in PBS-T (Tween_20_) (0.1%) with 1% BSA and incubated for 1.5 h at RT. The lectin solution was removed, and wells were washed three times with 300 µL of PBS-T; 100 µL of Streptavidin-HRP (1:7,000) (Invitrogen) in PBS-T with 1% BSA was added to wells, and the plate was incubated at RT for 1 h. The secondary solution was discarded, and wells were washed three times with 300 µL of PBS-T. Any remaining solution was removed from wells, and the plate was developed with 50 µL of 3,3′,5,5′-tetramethylbenzidine substrate. Plates were incubated at RT in the dark for 15 min, and 50 µL of 1 M hydrochloric acid was added to each well to stop the reaction. The optical density at 450 nm was measured using a plate reader with background corrections to each sample. The assay was performed in technical quadruplicates.

### Statistical analysis

One-way analysis of variances with Tukey’s post hoc test was used to compare SBA binding with the heptasaccharide glycan present in whole cell lysates across the different *C. hepaticus* HV10^T^ strains. Statistical analysis was performed using GraphPad Prism version 10.1.2.324 (Dotmatics).

## Data Availability

Sequence data from the plasmids in this study have been deposited in the GenBank Data Libraries under the accession numbers PP589919; pCC311, PP589920; pCC388, PP589921; pJBM2, PP589922; pJBM3, PP589923; pCH_pglB_SV, PP589924; pJBM4, PP589925; pJBM5.1, and PP589926; pJBM5.2. The sequence reads of *C. hepaticus* HV10^T^∆*pglB::kan* M7 and *C. hepaticus* HV10^T^∆*pglB::kan* M3 have been deposited in the Sequence Read Archive (SRA) under the accession numbers SRX24788994 and SRX24803174, respectively. The *C. hepaticus* HV10^T^∆*pglB::kan* mutant 7 genome assembly is deposited in GenBank under the accession number JBEFTY000000000.

## SUPPLEMENTAL MATERIAL

Supplemental material mbio.01101-24-s0001.docx Fig. S1-S8; Tables S1 and S2.

ASM does not own the copyrights to Supplemental Material that may be linked to, or accessed through, an article. The authors have granted ASM a non-exclusive, world-wide license to publish the Supplemental Material files. Please contact the corresponding author directly for reuse.

## References

[1] Quesada-Vásquez D, Jiménez-Madrigal L, Chaves-Hernández A, Muñoz-Vargas L, Barquero-Calvo E. 2023. First report of Campylobacter hepaticus isolation in laying hens and broiler breeders with spotty liver disease in Costa Rica. Avian Dis 67:89–93. doi:10.1637/aviandiseases-D-22-00046 37140116

[2] Gharbi M, Béjaoui A, Ben Hamda C, Alaya N, Hamrouni S, Bessoussa G, Ghram A, Maaroufi A. 2022. Campylobacter spp. in eggs and laying hens in the north-east of Tunisia: high prevalence and multidrug-resistance phenotypes. Vet Sci 9:108. doi:10.3390/vetsci9030108 35324836 PMC8952296

[3] Hananeh W, Ababneh M. 2021. Spotty liver disease in Jordan: an emerging disease. Vet Med 66:94–98. doi:10.17221/73/2020-VETMED PMC11975266 40201433

[4] Crawshaw T. 2019. A review of the novel thermophilic Campylobacter, Campylobacter hepaticus, a pathogen of poultry. Transbound Emerg Dis 66:1481–1492. doi:10.1111/tbed.13229 31081981

[5] Van TTH, Elshagmani E, Gor M-C, Anwar A, Scott PC, Moore RJ. 2017. Induction of spotty liver disease in layer hens by infection with Campylobacter hepaticus. Vet Microbiol 199:85–90. doi:10.1016/j.vetmic.2016.12.033 28110791

[6] Van TTH, Elshagmani E, Gor MC, Scott PC, Moore RJ. 2016. Campylobacter hepaticus sp. nov., isolated from chickens with spotty liver disease. Int J Syst Evol Microbiol 66:4518–4524. doi:10.1099/ijsem.0.001383 27498969

[7] Crawshaw T, Young S. 2003. Increased mortality on a free-range layer site. Vet Rec 153:664. 14667093

[8] Grimes T, Reece R. 2011. Spotty liver disease-an emerging disease in free range layers in Australia. Abstr Proc 16th Western Poultry Disease Conference; Sacramento, CA

[9] Crawshaw T, Irvine R. 2012. Spotty liver syndrome in poultry in great Britain. Vet Rec 170:317–318. doi:10.1136/vr.e2201 22447790

[10] Courtice JM, Mahdi LK, Groves PJ, Kotiw M. 2018. Spotty liver disease: a review of an ongoing challenge in commercial free-range egg production. Vet Microbiol 227:112–118. doi:10.1016/j.vetmic.2018.08.004 30473340

[11] Phung C, Scott PC, Dekiwadia C, Moore RJ, Van TTH. 2022. Campylobacter bilis sp. nov., isolated from chickens with spotty liver disease. Int J Syst Evol Microbiol 72:005314. doi:10.1099/ijsem.0.005314 35442881

[12] Van TTH, Phung C, Anwar A, Wilson TB, Scott PC, Moore RJ. 2023. Campylobacter bilis, the second novel Campylobacter species isolated from chickens with spotty liver disease, can cause the disease. Vet Microbiol 276:109603. doi:10.1016/j.vetmic.2022.109603 36423482

[13] Moore RJ, Scott PC, Van TTH. 2019. Spotlight on avian pathology: Campylobacter hepaticus, the cause of spotty liver disease in layers. Avian Pathol 48:285–287. doi:10.1080/03079457.2019.1602247 30942612

[14] Van TTH, Lacey JA, Vezina B, Phung C, Anwar A, Scott PC, Moore RJ. 2019. Survival mechanisms of Campylobacter hepaticus identified by genomic analysis and comparative transcriptomic analysis of in vivo and in vitro derived bacteria. Front Microbiol 10:107. doi:10.3389/fmicb.2019.00107 30804905 PMC6371046

[15] Walker RI, Caldwell MB, Lee EC, Guerry P, Trust TJ, Ruiz-Palacios GM. 1986. Pathophysiology of Campylobacter enteritis. Microbiol Rev 50:81–94. doi:10.1128/mr.50.1.81-94.1986 3515146 PMC373055

[16] Taylor DE. 1992. Genetics of Campylobacter and Helicobacter. Annu Rev Microbiol 46:35–64. doi:10.1146/annurev.mi.46.100192.000343 1444260

[17] Wassenaar TM, Fry BN, van der Zeijst BA. 1993. Genetic manipulation of Campylobacter: evaluation of natural transformation and electro-transformation. Gene 132:131–135. doi:10.1016/0378-1119(93)90525-8 8406035

[18] Labigne-Roussel A, Harel J, Tompkins L. 1987. Gene transfer from Escherichia coli to Campylobacter species: development of shuttle vectors for genetic analysis of Campylobacter jejuni. J Bacteriol 169:5320–5323. doi:10.1128/jb.169.11.5320-5323.1987 2822671 PMC213946

[19] Wang Y, Taylor DE. 1990. Natural transformation in Campylobacter species. J Bacteriol 172:949–955. doi:10.1128/jb.172.2.949-955.1990 2404960 PMC208523

[20] Yao R, Alm RA, Trust TJ, Guerry P. 1993. Construction of new Campylobacter cloning vectors and a new mutational cat cassette. Gene 130:127–130. doi:10.1016/0378-1119(93)90355-7 8344519

[21] Wösten MM, Boeve M, Koot MG, van Nuenen AC, van der Zeijst BA. 1998. Identification of Campylobacter jejuni promoter sequences. J Bacteriol 180:594–599. doi:10.1128/JB.180.3.594-599.1998 9457862 PMC106926

[22] Guerry P, Alm RA, Power ME, Logan SM, Trust TJ. 1991. Role of two flagellin genes in Campylobacter motility. J Bacteriol 173:4757–4764. doi:10.1128/jb.173.15.4757-4764.1991 1856171 PMC208154

[23] Wassenaar TM, Bleumink-Pluym NM, van der Zeijst BA. 1991. Inactivation of Campylobacter jejuni flagellin genes by homologous recombination demonstrates that flaA but not flaB is required for invasion. EMBO J 10:2055–2061. doi:10.1002/j.1460-2075.1991.tb07736.x 2065653 PMC452888

[24] Fry BN, Feng S, Chen YY, Newell DG, Coloe PJ, Korolik V. 2000. The galE gene of Campylobacter jejuni is involved in lipopolysaccharide synthesis and virulence. Infect Immun 68:2594–2601. doi:10.1128/IAI.68.5.2594-2601.2000 10768949 PMC97464

[25] Bachtiar BM, Coloe PJ, Fry BN. 2007. Knockout mutagenesis of the kpsE gene of Campylobacter jejuni 81116 and its involvement in bacterium-host interactions. FEMS Immunol Med Microbiol 49:149–154. doi:10.1111/j.1574-695X.2006.00182.x 17266722

[26] Talukdar PK, Negretti NM, Turner KL, Konkel ME. 2020. Molecular dissection of the Campylobacter jejuni CadF and FlpA virulence proteins in binding to host cell fibronectin. Microorganisms 8:389. doi:10.3390/microorganisms8030389 32168837 PMC7143056

[27] Dasti JI, Tareen AM, Lugert R, Zautner AE, Gross U. 2010. Campylobacter jejuni: a brief overview on pathogenicity-associated factors and disease-mediating mechanisms. Int J Med Microbiol 300:205–211. doi:10.1016/j.ijmm.2009.07.002 19665925

[28] Miller JF, Dower WJ, Tompkins LS. 1988. High-voltage electroporation of bacteria: genetic transformation of Campylobacter jejuni with plasmid DNA. Proc Natl Acad Sci U S A 85:856–860. doi:10.1073/pnas.85.3.856 3277182 PMC279654

[29] Luo N, Zhang Q. 2001. Molecular characterization of a cryptic plasmid from Campylobacter jejuni. Plasmid 45:127–133. doi:10.1006/plas.2000.1509 11322827

[30] Alfredson DA, Korolik V. 2003. Sequence analysis of a cryptic plasmid pCJ419 from Campylobacter jejuni and construction of an Escherichia coli-Campylobacter shuttle vector. Plasmid 50:152–160. doi:10.1016/s0147-619x(03)00060-x 12932741

[31] Szymanski CM, Yao R, Ewing CP, Trust TJ, Guerry P. 1999. Evidence for a system of general protein glycosylation in Campylobacter jejuni. Mol Microbiol 32:1022–1030. doi:10.1046/j.1365-2958.1999.01415.x 10361304

[32] Jervis AJ, Butler JA, Lawson AJ, Langdon R, Wren BW, Linton D. 2012. Characterization of the structurally diverse N-linked glycans of Campylobacter species. J Bacteriol 194:2355–2362. doi:10.1128/JB.00042-12 22389484 PMC3347071

[33] Nothaft H, Scott NE, Vinogradov E, Liu X, Hu R, Beadle B, Fodor C, Miller WG, Li J, Cordwell SJ, Szymanski CM. 2012. Diversity in the protein N-glycosylation pathways within the Campylobacter genus. Mol Cell Proteomics 11:1203–1219. doi:10.1074/mcp.M112.021519 22859570 PMC3494190

[34] Cain JA, Dale AL, Sumer-Bayraktar Z, Solis N, Cordwell SJ. 2020. Identifying the targets and functions of N-linked protein glycosylation in Campylobacter jejuni. Mol Omics 16:287–304. doi:10.1039/d0mo00032a 32347268

[35] Abouelhadid S, North SJ, Hitchen P, Vohra P, Chintoan-Uta C, Stevens M, Dell A, Cuccui J, Wren BW. 2019. Quantitative analyses reveal novel roles for N-glycosylation in a major enteric bacterial pathogen. mBio 10:e00297-19. doi:10.1128/mBio.00297-19 31015322 PMC6478998

[36] Cain JA, Dale AL, Niewold P, Klare WP, Man L, White MY, Scott NE, Cordwell SJ. 2019. Proteomics reveals multiple phenotypes associated with N-linked glycosylation in Campylobacter jejuni. Mol Cell Proteomics 18:715–734. doi:10.1074/mcp.RA118.001199 30617158 PMC6442361

[37] Szymanski CM, Burr DH, Guerry P. 2002. Campylobacter protein glycosylation affects host cell interactions. Infect Immun 70:2242–2244. doi:10.1128/IAI.70.4.2242-2244.2002 11895996 PMC127875

[38] McDonald JB, Scott NE, Underwood GJ, Andrews DM, Van TTH, Moore RJ. 2023. Characterisation of N-linked protein glycosylation in the bacterial pathogen Campylobacter hepaticus. Sci Rep 13:227. doi:10.1038/s41598-022-26532-0 36604449 PMC9816155

[39] Kelly J, Jarrell H, Millar L, Tessier L, Fiori LM, Lau PC, Allan B, Szymanski CM. 2006. Biosynthesis of the N-linked glycan in Campylobacter jejuni and addition onto protein through block transfer. J Bacteriol 188:2427–2434. doi:10.1128/JB.188.7.2427-2434.2006 16547029 PMC1428418

[40] Miller WG, Heath S, Mandrell RE. 2007. Cryptic plasmids isolated from Campylobacter strains represent multiple, novel incompatibility groups. Plasmid 57:108–117. doi:10.1016/j.plasmid.2006.08.005 17064774

[41] del Solar G, Giraldo R, Ruiz-Echevarría MJ, Espinosa M, Díaz-Orejas R. 1998. Replication and control of circular bacterial plasmids. Microbiol Mol Biol Rev 62:434–464. doi:10.1128/MMBR.62.2.434-464.1998 9618448 PMC98921

[42] Jesse TW, Pittenger-Alley LG, Englen MD. 2006. Sequence analysis of two cryptic plasmids from an agricultural isolate of Campylobacter coli. Plasmid 55:64–69. doi:10.1016/j.plasmid.2005.06.001 16120460

[43] Stoler N, Nekrutenko A. 2021. Sequencing error profiles of Illumina sequencing instruments. NAR Genom Bioinform 3:lqab019. doi:10.1093/nargab/lqab019 33817639 PMC8002175

[44] Heath RJ, Rubin JR, Holland DR, Zhang E, Snow ME, Rock CO. 1999. Mechanism of triclosan inhibition of bacterial fatty acid synthesis. J Biol Chem 274:11110–11114. doi:10.1074/jbc.274.16.11110 10196195

[45] Miller WG, Bates AH, Horn ST, Brandl MT, Wachtel MR, Mandrell RE. 2000. Detection on surfaces and in Caco-2 cells of Campylobacter jejuni cells transformed with new gfp, yfp, and cfp marker plasmids. Appl Environ Microbiol 66:5426–5436. doi:10.1128/AEM.66.12.5426-5436.2000 11097924 PMC92478

[46] Holt JP, Grant AJ, Coward C, Maskell DJ, Quinlan JJ. 2012. Identification of Cj1051c as a major determinant for the restriction barrier of Campylobacter jejuni strain NCTC11168. Appl Environ Microbiol 78:7841–7848. doi:10.1128/AEM.01799-12 22923403 PMC3485944

[47] Abouelhadid S, Raynes J, Bui T, Cuccui J, Wren BW. 2020. Characterization of posttranslationally modified multidrug efflux pumps reveals an unexpected link between glycosylation and antimicrobial resistance. mBio 11:e02604-20. doi:10.1128/mBio.02604-20 33203757 PMC7683400

[48] Cain JA, Dale AL, Cordwell SJ. 2021. Exploiting pglB oligosaccharyltransferase-positive and -negative Campylobacter jejuni and a multiprotease digestion strategy to identify novel sites modified by N-linked protein glycosylation. J Proteome Res 20:4995–5009. doi:10.1021/acs.jproteome.1c00482 34677046

[49] Karlyshev AV, Everest P, Linton D, Cawthraw S, Newell DG, Wren BW. 2004. The Campylobacter jejuni general glycosylation system is important for attachment to human epithelial cells and in the colonization of chicks. Microbiology (Reading) 150:1957–1964. doi:10.1099/mic.0.26721-0 15184581

[50] Wacker M, Linton D, Hitchen PG, Nita-Lazar M, Haslam SM, North SJ, Panico M, Morris HR, Dell A, Wren BW, Aebi M. 2002. N-linked glycosylation in Campylobacter jejuni and its functional transfer into E. coli. Science 298:1790–1793. doi:10.1126/science.298.5599.1790 12459590

[51] Van TTH, Lee Nen That LFM, Perera R, Anwar A, Wilson TB, Scott PC, Stanley D, Moore RJ. 2022. Spotty liver disease adversely affect the gut microbiota of layers hen. Front Vet Sci 9:1039774. doi:10.3389/fvets.2022.1039774 36387407 PMC9650437

[52] Linton D, Allan E, Karlyshev AV, Cronshaw AD, Wren BW. 2002. Identification of N-acetylgalactosamine-containing glycoproteins PEB3 and CgpA in Campylobacter jejuni. Mol Microbiol 43:497–508. doi:10.1046/j.1365-2958.2002.02762.x 11985725

[53] Korolik V, Moorthy L, Coloe PJ. 1995. Differentiation of Campylobacter jejuni and Campylobacter coli strains by using restriction endonuclease DNA profiles and DNA fragment polymorphisms. J Clin Microbiol 33:1136–1140. doi:10.1128/jcm.33.5.1136-1140.1995 7615717 PMC228118

[54] Wösten M. 1997. Initiation of transcription and gene organization in Campylobacter jejuni= Transcriptie-initiatie en genorganisatie in Campylobacter jejuni Ph.D. Thesis, Utrecht University, Netherlands

[55] Andrews S. 2010. FastQC: a quality control tool for high throughput sequence data. Available from: http://www.bioinformatics.babraham.ac.uk/projects/fastqc. Retrieved 15 Jun 2023.

[56] Bolger AM, Lohse M, Usadel B. 2014. Trimmomatic: a flexible trimmer for Illumina sequence data. Bioinformatics 30:2114–2120. doi:10.1093/bioinformatics/btu170 24695404 PMC4103590

[57] Antipov D, Hartwick N, Shen M, Raiko M, Lapidus A, Pevzner PA. 2016. plasmidSPAdes: assembling plasmids from whole genome sequencing data. Bioinformatics 32:3380–3387. doi:10.1093/bioinformatics/btw493 27466620

[58] Wick RR, Judd LM, Gorrie CL, Holt KE. 2017. Unicycler: resolving bacterial genome assemblies from short and long sequencing reads. PLoS Comput Biol 13:e1005595. doi:10.1371/journal.pcbi.1005595 28594827 PMC5481147

[59] Afgan E, Nekrutenko A, Grüning BA, Blankenberg D, Goecks J, Schatz MC, Ostrovsky AE, Mahmoud A, Lonie AJ, Syme A, et al.. 2022. The galaxy platform for accessible, reproducible and collaborative biomedical analyses: 2022 update. Nucleic Acids Res 50:W345–W351. doi:10.1093/nar/gkac247 35446428 PMC9252830

[60] Sayers EW, Bolton EE, Brister JR, Canese K, Chan J, Comeau DC, Connor R, Funk K, Kelly C, Kim S, Madej T, Marchler-Bauer A, Lanczycki C, Lathrop S, Lu Z, Thibaud-Nissen F, Murphy T, Phan L, Skripchenko Y, Tse T, Wang J, Williams R, Trawick BW, Pruitt KD, Sherry ST. 2022. Database resources of the national center for biotechnology information. Nucleic Acids Res 50:D20–D26. doi:10.1093/nar/gkab1112 34850941 PMC8728269

[61] Coil D, Jospin G, Darling AE. 2015. A5-miseq: an updated pipeline to assemble microbial genomes from Illumina MiSeq data. Bioinformatics 31:587–589. doi:10.1093/bioinformatics/btu661 25338718

[62] Gibson DG, Young L, Chuang R-Y, Venter JC, Hutchison CA III, Smith HO. 2009. Enzymatic assembly of DNA molecules up to several hundred kilobases. Nat Methods 6:343–345. doi:10.1038/nmeth.1318 19363495

[63] Solovyev ASV. 2011. Automatic annotation of microbial genomes and metagenomic sequences. In Metagenomics and its applications in agriculture, biomedicine and environmental studies. Nova Science Publishers.

