# Supplementary material for: Development of tools for the genetic manipulation of Campylobacter and their application to the N-glycosylation system of Campylobacter hepaticus, an emerging pathogen of poultry
Source: mBio. 2024 Jul 29;15(9):e01101-24. doi: 10.1128/mbio.01101-24 (PMC11389370; doi:10.1128/mbio.01101-24)
Supplement: Supplemental material — Fig. S1-S8; Tables S1 and S2. [file mbio.01101-24-s0001.docx]

**Supplementary Material**


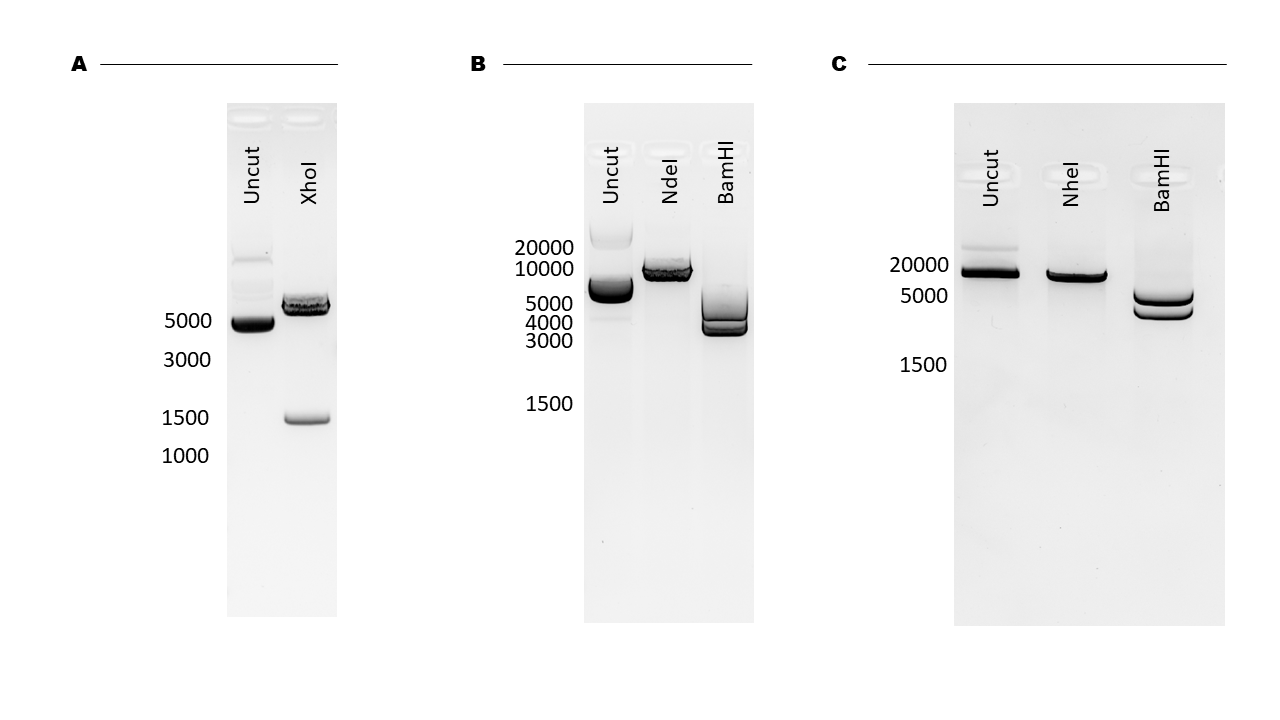
**Fig. S1.** Diagnostic restriction digest of shuttle vectors. (A) diagnostic digest of pJBM1 isolated from *E. coli* NEB5-alpha. *Xho*I drops out the insert (1,274 bp) from the backbone (4,355 bp). (B) Diagnostic digest of pJBM2 isolated from *C. hepaticus* HV10^T^. *Nde*I linearizes the plasmid within the insert (cryptic plasmid amplicon) and *BamH*I cuts the vector twice producing band sizes of 4,137 bp and 2,958 bp. (C) Diagnostic digest of pJBM3 isolated from *C. hepaticus* HV10^T^. *Nhe*I linearizes the plasmid within the insert (cryptic plasmid amplicon) and *BamH*I cuts the vector twice producing band sizes of 4,695 bp and 2,958 bp.


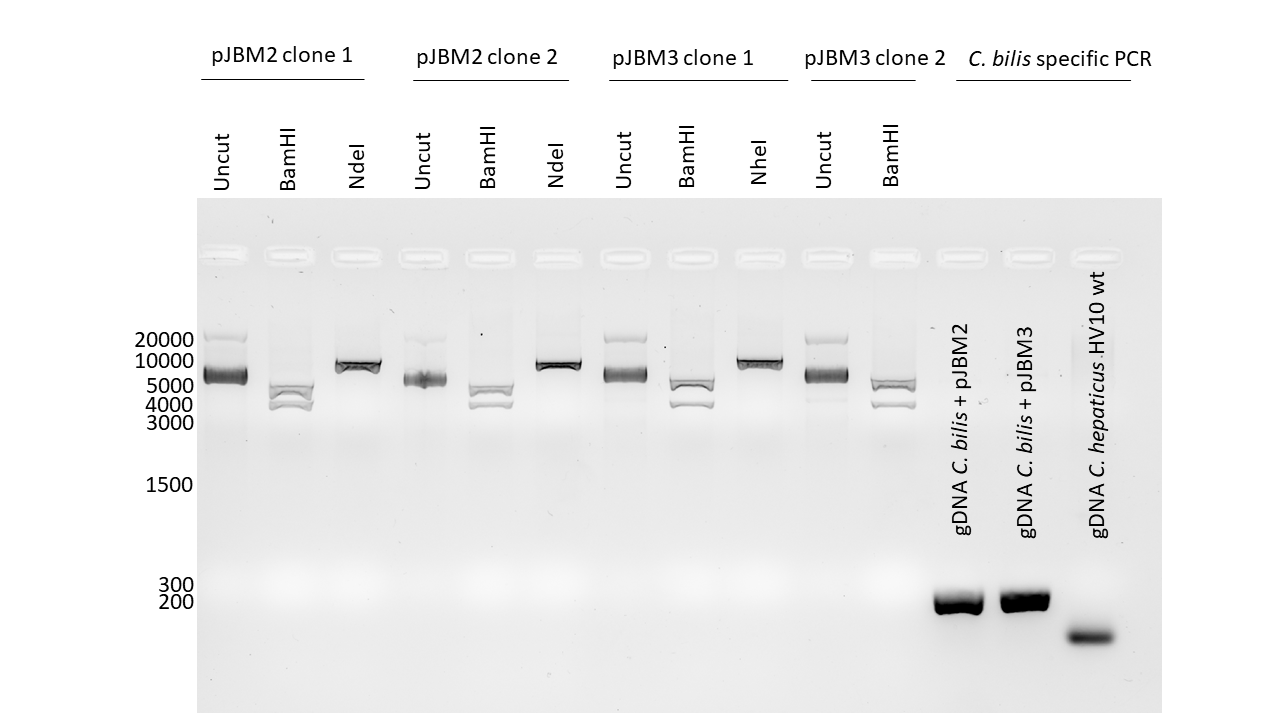


**Fig. S2.** Diagnostic restriction digest of shuttle vectors isolated from *C. bilis* VicNov18^T^ and PCR to confirm tranfromants were *C. bilis*. Lanes 1-6: diagnostic digests of pJBM2 isolated from two different clones of *C. bilis* transformed with pJBM2. *Nde*I linearizes pJBM2 within the insert (cryptic plasmid amplicon) and *BamH*I cuts the vector twice producing band sizes of 4,137 bp and 2,958 bp. Lanes 7-11: diagnostic digest of pJBM3 isolated from two different clones of *C. bilis* transformed with pJBM3. *Nhe*I linearizes pJBM3 within the insert (cryptic plasmid amplicon) and *BamH*I cuts the vector twice producing band sizes of 4,695 bp and 2,958 bp. Lanes 12-14: *C. bilis* specific PCR using genomic DNA from *C. bilis* transformed with pJBM2 and pJBM3 as a template. PCR was performed using primer pairs QLD2F2 and QLD2R1 as described in (1) producing a 258 bp product, which is lacking in the PCR using *C. hepaticus* HV10^T^ genomic DNA as a template (negtive control).


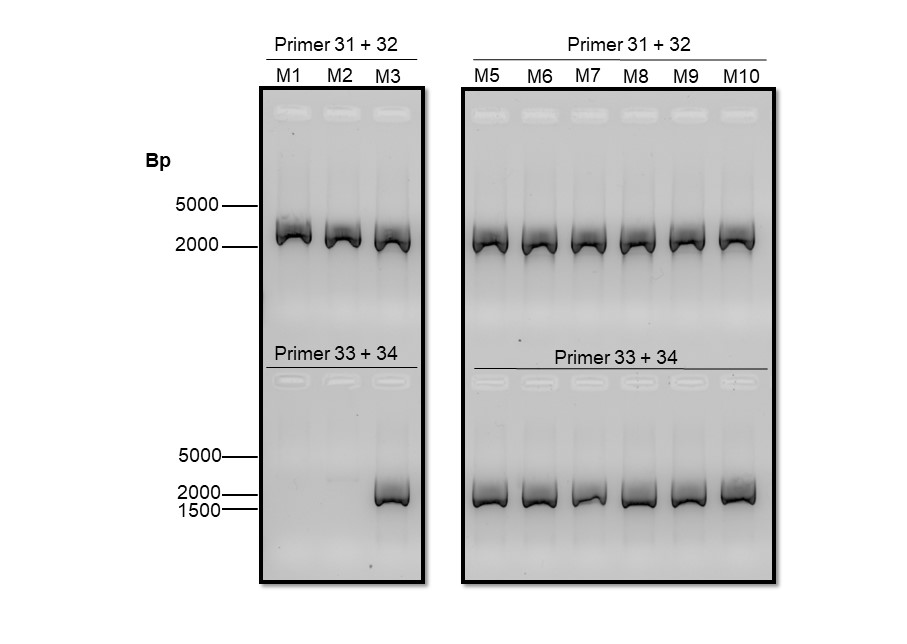


**Fig. S3.** PCR to confirm *C. hepaticus* HV10^T^*∆pglB*::*kan* double cross-over recombinant mutants. *C. hepaticus* HV10^T^*∆pglB*::*kan* mutants 1,2, 3 and are independently derived from mutants 5, 6, 7, 8, 9, 10. Primers 31 + 32 produce a 2240 bp product. Primers 33 + 34 produce a 1,817bp product. *C. hepaticus* HV10^T^ gDNA was used as a negative control for amplification for both sets of primers. In each case no product was observed (not shown). Results of the PCR indicate Mutants 1 and 2 are single cross-over recombinants and mutants 3, 5, 6, 7, 8, 9 and 10 are double cross-over recombinants.


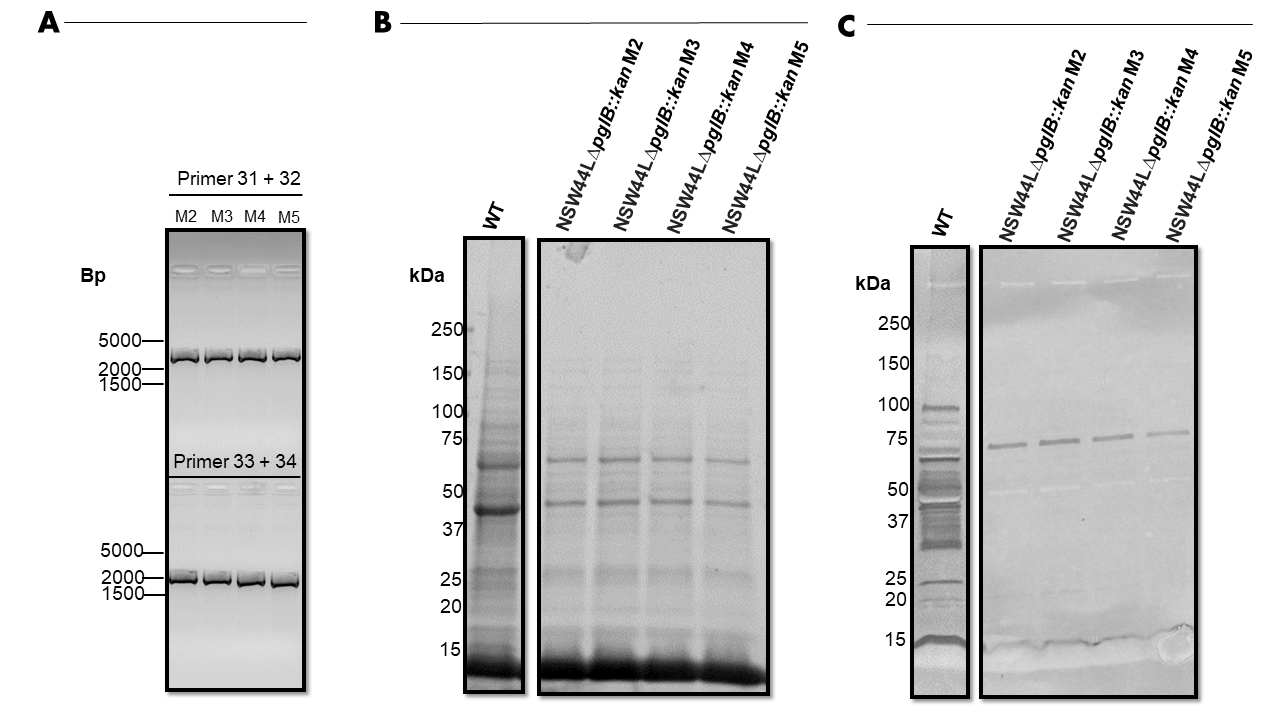


**Fig. S4.** Site-directed mutagensis of *pglB* in *C. hepaticus* NSW44L. (A) PCR to confirm *C. hepaticus* NSW44L*∆pglB*::*kan* double cross-over recombinant mutants. Primers 31 + 32 produce a 2,240 bp product. Primers 33 + 34 produce a 1,817bp product. *C. hepaticus* NSW44L gDNA was used as a negative control for amplification for both sets of primers. In each case no product was observed (not shown). (B) (SDS-PAGE) and (C) Soybean agglutinin lectin blotting of C. hepaticus NSW44L (WT) and C. hepaticus NSW44L*∆*pglB::kan mutants of whole cell lysates containing 33-37 μg of protein. Whole cell lysates were separated by 8-16% SDS-PAGE and either developed with SimplyBlue™ SafeStain or transferred to PVDF membranes for lectin blotting.


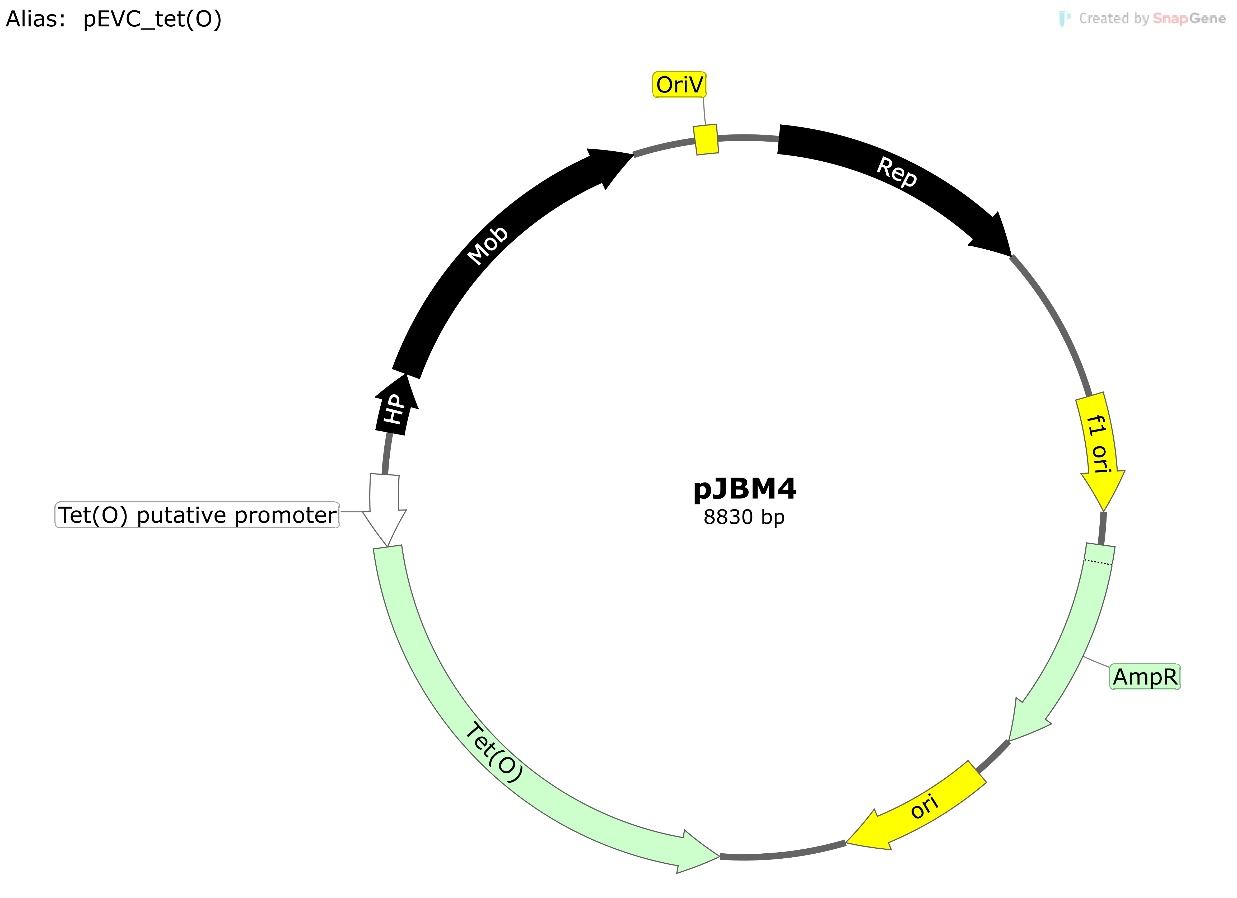


**Fig. S5.** Plasmid map of pJBM4 sequenced shuttle vector, which served as an empty vector control.


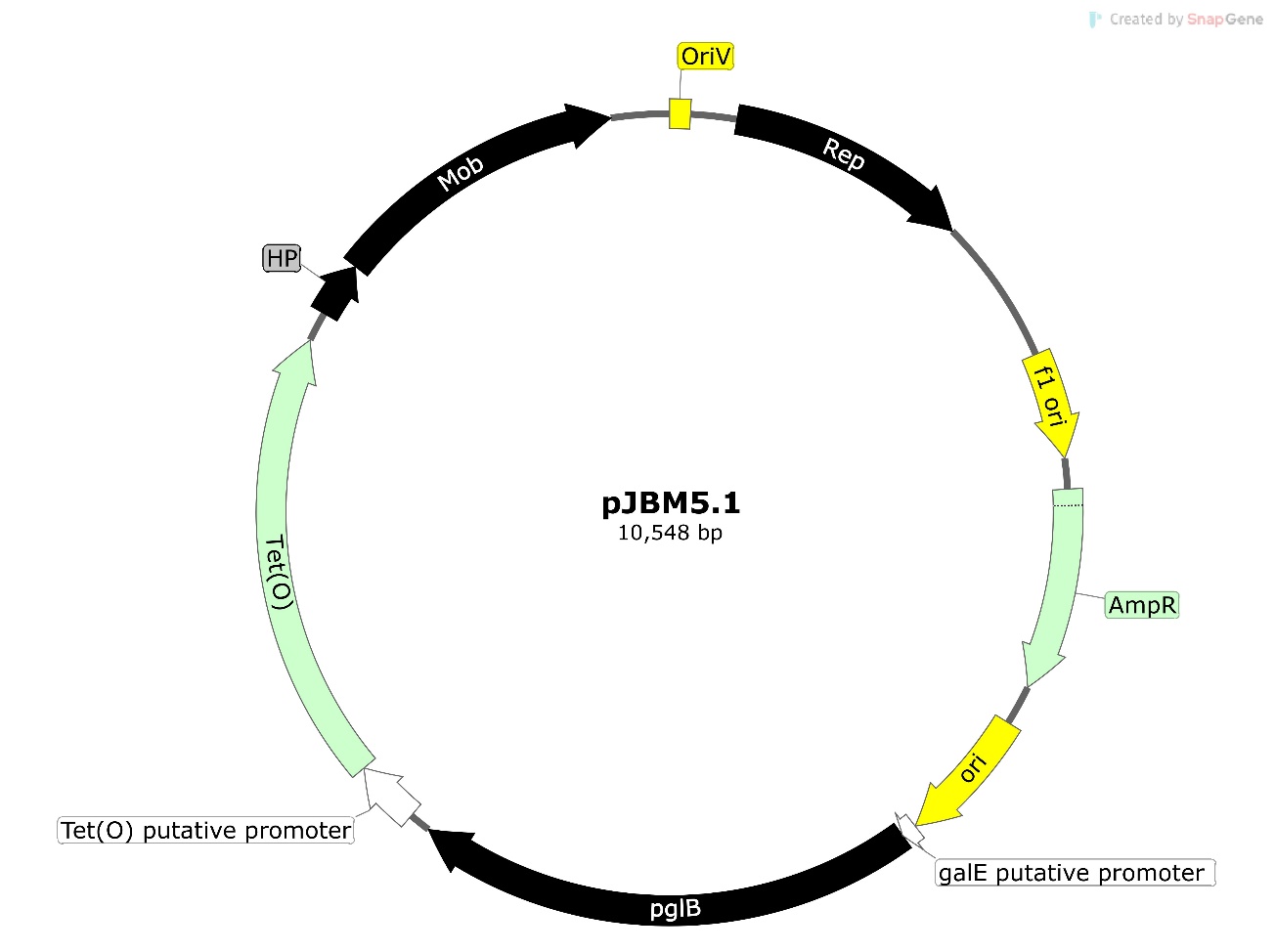


**Fig. S6.** Plasmid map of sequenced pJBM5.1 *pglB* expression vector


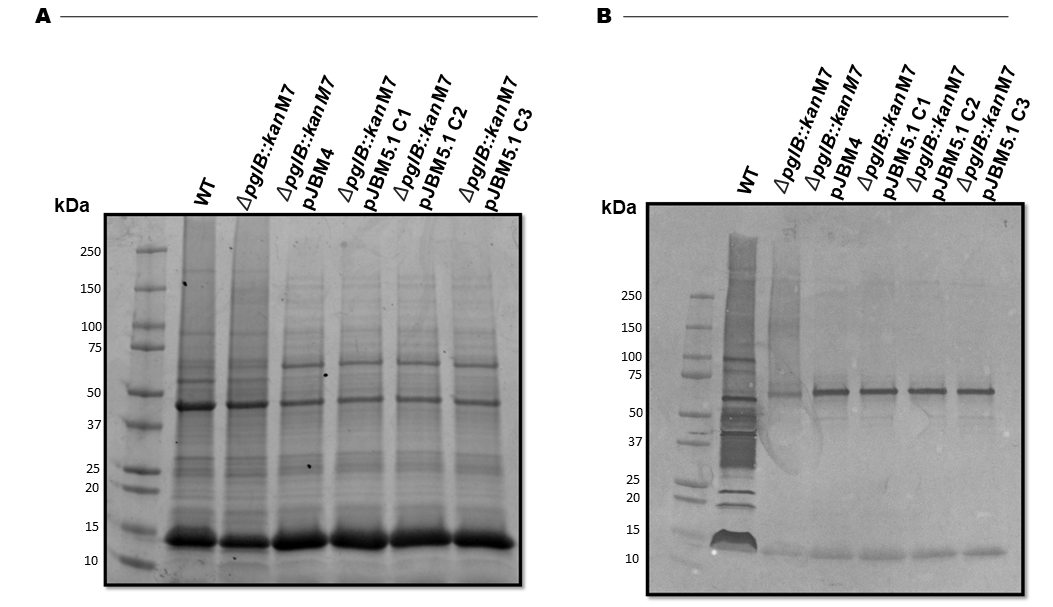


**Fig. S7.** Complementation of *C. hepaticus* HV10^T^*∆*pglB::kan mutant 7 in trans with pJBM5.1, encoding PglB. (A) SDS-PAGE, (B) SBA lectin blot of *C. hepaticus* HV10^T^ (WT), *C. hepaticus* HV10^T^*∆pglB::kan* mutant 7, *C. hepaticus* HV10^T^*∆pglB::kan* mutant 7 (pJBM4), and three separate (C1, C2, C3) *C. hepaticus* HV10^T^*∆pglB::kan* mutant 7 clones expressing the functional *pglB* encoded on pJBM5.1 whole cell lysates containing 20 -25 µg of protein. Whole cell lysates were separated by 4–20% SDS-PAGE and either developed with SimplyBlue™ SafeStain or transferred to PVDF membranes for lectin blotting.


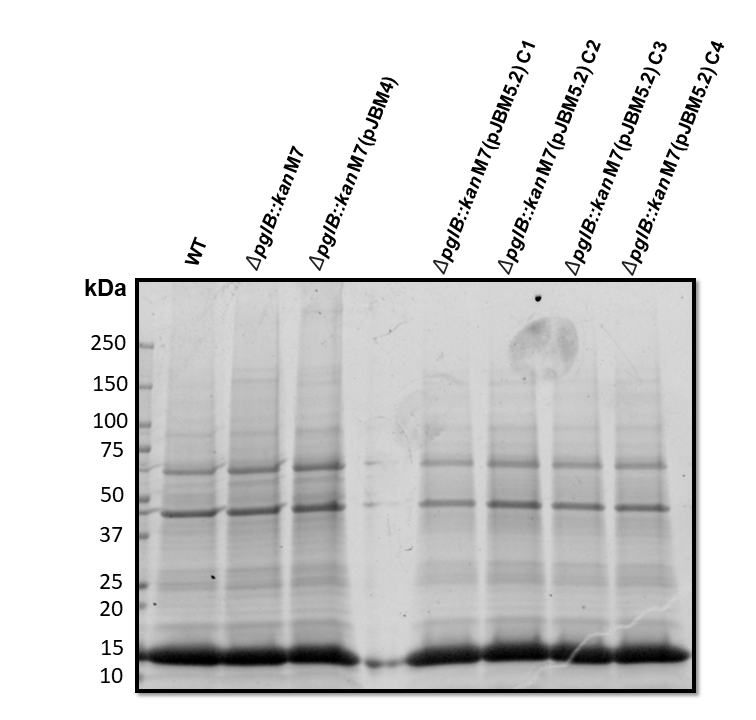


**Fig S8.** Complementation of *C. hepaticus* HV10^T^*∆pglB::kan* mutant 7 *in trans* with pJBM5.2, encoding *pglB* and all genes downstream of *pglB* in the *pgl* locus, excluding *pglG*. SDS-PAGE of *C. hepaticus* HV10^T^ *C. hepaticus* HV10^T^*∆pglB::kan* mutant 7, *C. hepaticus* HV10^T^*∆pglB::kan* mutant 7(pJBM4) (empty vector control), and two clones from separate isogenic complements (clones 1 and 2 were derived independently of clones 3 and 4) of *C. hepaticus* HV10^T^*∆pglB::kan* mutant 7 clones expressing functional *pgl* genes encoded on pJBM5.2. Whole cell lysates contained 27 μg of protein and were separated by 4–20% SDS-PAGE and developed with SimplyBlue™ SafeStain.

**Table S1.** Tukey’s multiple comparisons test of ELISA results of SBA binding to whole cell lysates of *C. hepaticus* HV10^T^, *C. hepaticus* HV10^T^*∆pglB::kan* mutant 7, *C. hepaticus* HV10^T^*∆pglB::kan* mutant 7(pJBM4), *C. hepaticus* HV10^T^*∆pglB::kan* mutant 7(pJBM5.2), and *Salmonella* Typhimurium 82/6915*∆aroA* (STM1) (negative control).


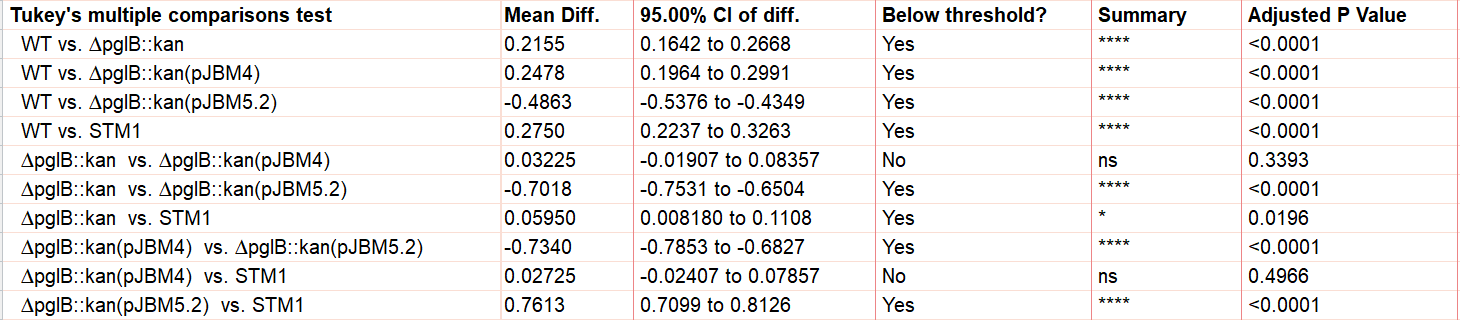


**Table S2. Primers used in the study**

| **Target** | **Template** | **Orientation** | **Primer name & sequence (5’🡪3’)** | **Amplicon size (bp)** |
| --- | --- | --- | --- | --- |
| Replication elements from pWestmill 11 for pJBM1 | pWestmill 11 | F | Primer 1: CCACTCGAGGGACAAGACACCGCCCTATT | 1,413 |
|  |  | R | Primer 2:  ACTCTCGAGCGTTAGCTCTAGGCTAACTTC |  |
| Linearization of pMW2 for pJBM2 | pMW2 | F | Primer 3: AACTTTGTATTTGTATAGCA  GCAAGTTTTTATGCTTTGCCCA | 4,395 |
|  |  | R | Primer 4: GAGTAATAAGACAGGATATG  ATGGACTAATGCTTGAAACCCAG |  |
| Replication elements from pCC311 for pJBM2 | pCC311 | F | Primer 5: GGTTTCAAGCATTAGTCCATCATATCC  TGTCTTATTACTCCAAGCAATTATAATAAAAT | 2,780 |
|  |  | R | Primer 6: GGCAAAGCATAAAAACTTGCTGCTA  TACAAATACAAAGTTTTATCGCTAAAGC |  |
| Linearization of pMW2 for pJBM3 | pMW2 | F | Primer 7: GTGCCAAGTGTCGAGCTTG  TGCAAGTTTTTATGCTTTGCCCAT | 4,395 |
|  |  | R | Primer 8: GTGGGGTTAAGGGGTTGCAC  ATGGACTAATGCTTGAAACCCAG |  |
| Replication elements from pCC388 for pJBM3 | pCC388 | F | Primer 9: GGTTTCAAGCATTAGTCC  ATGTGCAACCCCTTAACCCC | 3,338 |
|  |  | R | Primer 10: GGCAAAGCATAAAAACTTG  CACAAGCTCGACACTTGGC |  |
| Linearization of pJBM3 for Insertion of *tet(O)* gene for pJBM4 | pJBM3 | F | Primer 11: TCAAGCTGACAAACTAAAGCCTGGGTTTCAAGCATTAGT | 6,626 |
|  |  | R | Primer 12: TGTTCCACAAGTTAGCTTAAGGGATCCACTAGTTCTAGAGCG |  |
| *tet^R^* and its putative promoter for insertion into pJBM3 for pJBM4 | *C. hepaticus* 84B pDNA | F | Primer 13: CTCTAGAACTAGTGGATCCCTTA  AGCTAACTTGTGGAACATATGCC | 2,284 |
|  |  | R | Prime 14: GACTAATGCTTGAAACCCAGGCTTTAGTTTGTCAGCTTGACAAA |  |
| Linearization of pMW2 for pCH_*pglB*_SV | pMW2 | F | Primer 15: CACTTTATGATGAAGAGGTA  ACTGGCCGTCGTTTTACAAC | 3,007 |
|  |  | R | Primer 16:  TGCTAGTTTTTGCATAAAGCCGGCCGCCACCG |  |
| Left flanking region of *pglB* for CH_*pglB*_SV | *C. hepaticus* HV10^T^ gDNA | F | Primer 17: CTCCACCGCGGTGGCGGCCGGCTTTATGCAAAAACTAGCAATTTTTATTTATTCT | 1,143 |
|  |  | R | Primer 18: ATGCAAGTTTTTATGCTTTGTTACCCTAATAA  ATATTTCAAAGCATCACGTGC |  |
| *aph(3')-IIIa* gene for CH_*pglB*_SV | pMW2 | F | Primer 19: TGAAATATTTATTAGGGTAACAAAGCATAAAAACTTGCATGGACTAAT | 1,150 |
|  |  | R | Primer 20: ATAAAAAACCTATTCTCATTCCCCGGGCTGCAG |  |
| Right flanking region *pglB* for CH_*pglB*_SV | *C. hepaticus* HV10^T^ gDNA | F | Primer 21: CGAATTCCTGCAGCCCGGGGAA  TGAGAATAGGTTTTTTATCGCACG | 1,160 |
|  |  | R | Primer 22: GTTGTAAAACGACGGCCAGTTACCTC  TTCATCATAAAGTGCTAAGTATTCTTG |  |
| Linearization of pJBM3 for pJBM5 | pJBM3 | F | Primer 23: TGTTCCACAAGTTAGCTTAACCTGGGTTTCAAGCATTAGT | 6,135 |
|  |  | R | Primer 24: TATCAAAAAGTTCAAAAAA  GCGTTTTTCCATAGGCTCCG |  |
| Intergenic region upstream of *pgl* locus for pJBM5 | *C. hepaticus* HV10^T^ gDNA | F | Primer 25: GCGGAGCCTATGGAAAAACGCTTTTTT  GAACTTTTTGATAAATTGTAAAACTGCTG | 198 |
|  |  | R | Primer 26: TTTAAATATTCTCTTTTTAACATTTTTTAACC  TTTTTTTAAATAATTTTGCTAAAATCATATCAGA |  |
| *pglB* for pJBM5 | *C. hepaticus* HV10^T^ gDNA | F | Primer 27: TAAAAAAAGGTTAAAAAATGTTAAAAAGAG  AATATTTAAAAAATCCATACTTACTTTTAGG | 2,176 |
|  |  | R | Primer 28: ATCATCTGTTTATGCAAAAATTAAATTTTAAGTTTAAAAACTTTAGCATCTTTAGAATTAAGAACT |  |
| *Tet^R^* for pJBM5 | *C. hepaticus* 84B pDNA | F | Primer 29: TTTTTAAACTTAAAATTTAATTTTT  GCATAAACAGATGATTAGTGGC | 2,336 |
|  |  | R | Primer 30: ACTAATGCTTGAAACCCAGGTT  AAGCTAACTTGTGGAACATATGC |  |
| Double homologous recombination Left flanking region screening primer 1 | *C. hepaticus* HV10^T^ *pglB::kan* gDNA | F | Primer 31: GGCAGCATACTATGCAAAAC | 2,240 |
|  |  | R | Primer 32: GCTCGACATACTGTTCTTCC |  |
| Double homologous recombination Right flaking region screening primer 2 | *C. hepaticus* HV10^T^ *pglB::kan* gDNA | F | Primer 33: GCTCGGAAGAGTATGAAGAT | 1,817 |
|  |  | R | Primer 34: CGTTCCTGAGTAAAGACCAC |  |
| Linearization of pJBM5 for pJBM5.1 | pJBM5 | F | Primer 35: TTTAAGATAAGGAAAGATTAATGTTAAAAAGAGAATATTTAAAAAATCCATACTTACTTTTAGG | 10,522 |
|  |  | R | Primer 36: ATTTTTTTTAATATTCAAGATTTCCATAGGCTCCGCC |  |
| Amplification of putative *galE* promoter for pJBM5.1 | *C. hepaticus* HV10^T^ gDNA | F | Primer 37: GGGGGCGGAGCCTATGGAAATCTTGAATATTAAAAAAAATATATTAAAATTTCTTTTAGTCTAAATT | 106 |
|  |  | R | Primer 38:  AAATATTCTCTTTTTAACATTAATCTTTCCTTATCTTAAAATTTAGACTAAAAGAAATTTTAATATATTT |  |
| Linearization of pJBM5 for pJBM5.2 | pJBM5 | F | Primer 39: TAATAAAGAAGGAATATAAATTTTGCATAAACAGATGATTAGTGGCAG | 8,426 |
|  |  | R | Primer 40: ATTTTTTTTAATATTCAAGATTTCCATAGGCTCCGCC |  |
| Amplification of putative *galE* promoter for pJBM5.2 | *C. hepaticus* HV10^T^ gDNA | F | Primer 41: GGGGGCGGAGCCTATGGAAATCTTGAATATTAAAAAAAATATATTAAAATTTCTTTTAGTCTAAATT | 106 |
|  |  | R | Primer 42: TATTCTCTTTTTAACATTTTTAATCTTTCCTTATCTTAAAATTTAGACTAAAAGAAATTT |  |
| Amplification of *pglB, pglA, pglC, pglD, pglE* and *pglF* for pJBM5.2 | *C. hepaticus* HV10^T^ gDNA | F | Primer 43: TTTAAGATAAGGAAAGATTAAAAATGTTAAAAAGAGAATATTTAAAAAATCCATACTTACTTTTAGG | 7,526 |
|  |  | R | Primer 44: AATCATCTGTTTATGCAAAATTTATATTCCTTCTTTATTATGCTTAAATTCAGGAACA |  |

References

1. Van TTH, Phung C, Anwar A, Wilson TB, Scott PC, Moore RJ. 2023. 808 *Campylobacter bilis*, the second novel *Campylobacter* species isolated from 809 chickens with Spotty Liver Disease, can cause the disease. Vet Microbiol 810 276:109603.
